# Unravelling long-term impact of water abstraction and climate change on endorheic lakes: A case study of Shortandy Lake in Central Asia

**DOI:** 10.1371/journal.pone.0305721

**Published:** 2024-07-18

**Authors:** Marzhan Baigaliyeva, Nick Mount, Simon N. Gosling, Suzanne McGowan

**Affiliations:** 1 School of Mining and Geosciences, Nazarbayev University, Astana, Kazakhstan; 2 School of Geography, University of Nottingham, Nottingham, United Kingdom; 3 Department of Aquatic Ecology, Netherlands Institute of Ecology, Wageningen, Netherlands; UNITEN: Universiti Tenaga Nasional, MALAYSIA

## Abstract

Endorheic lakes, lacking river outflows, are highly sensitive to environmental changes and human interventions. Central Asia (CA) has over 6000 lakes that have experienced substantial water level variability in the past century, yet causes of recent changes in many lakes remain unexplored. Modelling hydrological processes for CA lakes poses challenges in separating climatic change impacts from human management impacts due to limited data and long-term variability in hydrological regimes. This study developed a spatially lumped empirical model to investigate the effects of climate change and human water abstraction, using Shortandy Lake in Burabay National Nature Park (BNNP) as a case study. Modelling results show a significant water volume decline from 231.7x10^6^m^3^ in 1986 to 172.5x10^6^m^3^ in 2016, primarily driven by anthropogenic water abstraction, accounting for 92% of the total volume deficit. The highest rates of water abstraction (greater than 25% of annual outflow) occurred from 1989 to 1993, coinciding with the driest period. Since 2013, the water volume has increased due to increased precipitation and, more importantly, reduced water abstraction. Despite limited observational data with which to calibrate the model, it performs well. Our analysis underscores the challenges in modelling lakes in data-sparse regions such as CA, and highlights the importance and benefits of developing lake water balance models for the region.

## Introduction

The CA region comprises around a third of the world’s arid areas [1]. The region is characterised by high spatio-temporal variability in temperature and precipitation [2] and freshwater lakes are a critical source of water for ecosystems and population. Competition for water to support economic development is often cited as the primary driver of the demise of several major endorheic lakes in the region, including the Aral Sea and Lake Balkhash [3, 4], but climate change is also recognised as being an important factor [5–7]. A lack of success in balancing the needs of water for economic development versus ecosystem maintenance and health, and generally low levels of water efficiency, underpin the crisis facing CA lakes. High levels of surface and ground water abstraction to support agriculture (more than 90% of the region’s water supply is used to irrigate crops [8]), and economic development remain significant issues, which are often cited as being of primary concern [9]. Future climate scenarios for CA project rising air temperatures at a rate of 0.37°C per decade [10] and changes in precipitation and snow patterns [11, 12], which are expected to contribute to water scarcity in the region [13].

To ensure future water security, a detailed understanding of climatic and anthropogenic drivers of water scarcity is essential due to uncertainty surrounding freshwater availability and factors influencing water availability. Previous research has attributed lake volume changes in Kazakhstan primarily to climate variability rather than human activities (i.e. water abstraction) [14, 15], but contemporary thinking in the wider CA region challenges this perspective [16–18]. Therefore, it is unclear whether addressing climate or human impacts should be the primary focus for water management practices in Kazakhstan.

Endorheic lakes, which have no outflow, are critical water resources in the region and yet are hydrologically sensitive and vulnerable to climate change [19]. CA endorheic lakes provide a valuable focus for study. They provide a closed system for hydrological analysis that provides opportunity in unravelling the relative impacts of climate and anthropogenic activity. They are highly sensitive to changes in the different components of their overall water balance [19] which has resulted in long-term water volume variability [16, 20].

To evaluate water resources, it is crucial to measure and analyse the quantities and patterns of water movement and storage changes across large areas across in both spatial and temporal dimensions. Among the challenges in developing hydrological models for endorheic lakes worldwide is the scarcity of data and lack of field-based measurements [21]. The significance of on-site measurements and comprehensive modelling has been demonstrated in endorheic lake systems, such as Lake Chad [22], Lake Urmia [23], and the Aral Sea [18]. These factors contribute to the complexity of hydrological models and affect their accuracy [23, 24]. For CA lakes, environmental monitoring data is generally limited to a few sub-regions, primarily urban and industrial centres. The dissolution of the Soviet Union resulted in the discontinuation of many monitoring stations, particularly in remote areas [25]. This has led to insufficient monitoring of important water balance components, including precipitation [26, 27] and streamflow [28], and is considered a crucial factor hindering global research efforts to identify areas experiencing significant changes in water resources [29]. Additionally, restrictive data policies, especially in transboundary basins further compound these challenges [30]. These constraints pose significant difficulties in modelling hydrological processes in CA lakes and distinguishing between climatic changes and human interventions.

Here we adopt a spatially lumped empirical modelling approach to quantify the relative importance of climatic and anthropogenic water abstraction by analysing the historical patterns of change that have occurred in Shortandy Lake, a small endorheic lake in Burabay National Nature Park (BNNP) in the north of Kazakhstan. This study examines the difficulties and implications of developing hydrological models for endorheic lakes, using the case study of Shortandy to illustrate the challenges associated with incomplete data, highlighting the importance and potential usefulness of such models despite limited observational data with which to calibrate them.

## Study area and materials

### Study area

**Fig 1:**
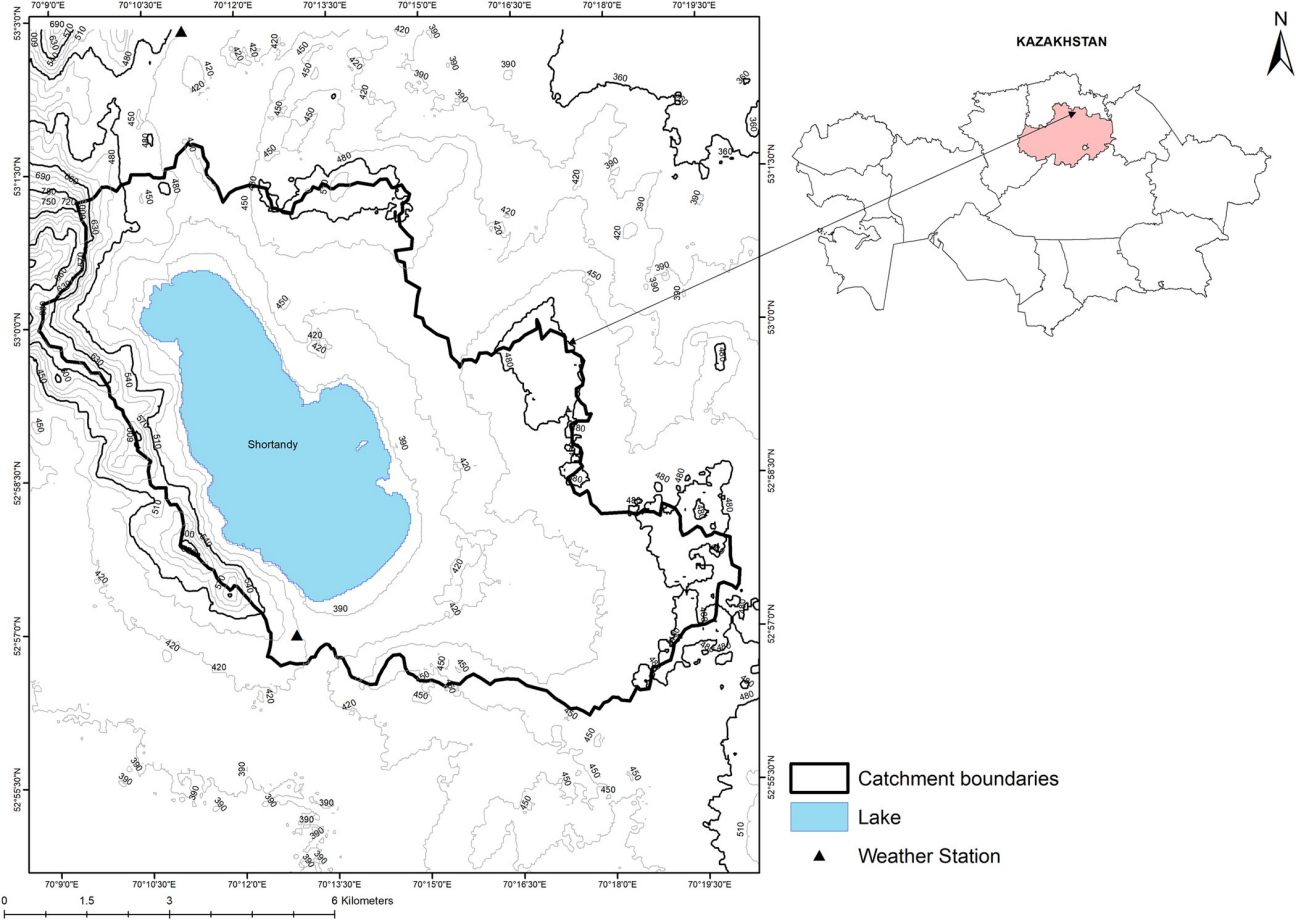
Location of the study area. The administrative boundary shapefile of Kazakhstan and its districts is obtained from an open license source known as the geoBoundaries Global Administrative Database [37].

BNNP has a total area of 1,296 km^2^ and consists of nearly 30 lakes which are mostly endorheic. Shortandy Lake (52°59′N, 70°13′E, 398m a.s.l.) is the largest endorheic lake within the BNNP (Fig 1) with a catchment area of 69.15 km^2^ (Table 1). The river network is sparse and most rivers and streams are fed by melting snow during the spring [31]. The Kylshakty River formerly drained Shortandy Lake until 1920, when the lake level dropped below 408m [14]. Shortandy Lake levels have fluctuated over the 20^th^ century, from a maximum of 408m a.s.l. between 1900–1920 [14] followed by a declining trend [32–35], with the maximum lake depth decreasing from 31m in 1956 to 23m in 2014.

**Table 1:** Physical characteristics of Shortandy Lake and local meteorological variables.

Feature	Value	Unit	Source
Lake area	15.7	km^2^	Landsat 8
Lake level	388.9	m a.s.l.	KazHydroMet, 2007
Maximum depth	22.7	m	Bathymetric survey in 2014
Total catchment area	69.15	km^2^	
Mean annual temperature	1.9	°C	From 1986–2016 in Schuchinsk weather station
Mean temperature (Nov-Apr)	-9.3	°C	From 1986–2016 in Schuchinsk weather station
Mean temperature (May-Oct)	13.1	°C	From 1986–2016 in Schuchinsk weather station
Mean wind speed	2.7	m/s	From 1986–2016 in Schuchinsk weather station
Mean annual precipitation	332	mm/yr	From 1986–2016 in Schuchinsk weather station
Mean precipitation (Nov-Apr)	77	mm/yr	From 1986–2016 in Schuchinsk weather station
Mean precipitation (May-Oct)	255	mm/yr	From 1986–2016 in Schuchinsk weather station

Similar to most lakes in the BNNP, Shortandy Lake is a tectonic lake that lies at the foot of the Kokshetau mountain ridge oriented from the north-west to the south-east. The lake was formed at the beginning of the Holocene in an aeolian depression [36]. The catchment orography is flat steppe and forested hills, where the lowest part (388m) is the lake and the highest area is 713m a.s.l. Vegetation cover is currently boreal forest with pine (65%), birch (31%), aspen (3%), and shrubs (1%) [31].

The lake shore is formed by Quaternary eluvial-deluvial deposits and sandy lacustrine sediments [36]. Most of the lake bottom is represented by sand and silt, and the central part is gyttja. The catchment is underlain by Ordovician sedimentary rocks (limestones, marls, argillites). The lake bed and local groundwater have good connectivity owing to the high permeability of the fractured zone [38]. Shallow groundwater provides water for boreal forest transpiration during dry summers and recharges mainly by snowmelt [38]. The major proportion of snow accumulates in the forested areas and eventually recharges groundwater storage by the end of the snowmelt season [39].

The climate of Shortandy is characterised by significant seasonal variability in air temperature, with cold season temperatures mostly below zero and ice cover on the lake surface (Table 1). The warm season arrives around March to April with subsequent snowmelt and ice-break-up on the lake. Total annual precipitation is on average 332mm, with 70% of the precipitation falling as rain (Table 1), but, the warm seasons are relatively dry, and the lake net balance is negative during these seasons.

Historically, the water level regime is characterised by so-called wet-dry periods where precipitation varies between years [32]. Previous water balance studies show that Shortandy’s water comes from surface runoff during snowmelt which varies annually and results in water level variability between 1-6m, whereas water output is mostly driven by open water evaporation [32, 35].

#### Water abstraction

BNNP is a major tourist attraction in Kazakhstan and Shortandy Lake is one of the most visited places with the largest settlement in the area (45,000 population). Water supply for domestic water consumption and recreation derives from surface and groundwater abstraction from several of the BNNP lakes. Water abstraction from Shortandy Lake has been the greatest of all the BNNP lakes [39]. Since the establishment of BNNP in 2000, the rapid development of tourism created a higher demand for water, especially during warm periods. Since 2010, declining water levels in Shortandy Lake prompted the government to limit water abstraction to 0.5 million m^3^ annually from the lake. Currently, Shortandy has a centralised water supply and both surface and groundwater abstraction barely exceeds the threshold of 0.5 million m^3^.

### Data sources

#### Meteorological data

Mean daily meteorological data (1986–2016) were obtained from the only local weather station in the study area (Fig 1, Table 1). Missing parameters required for open lake evaporation and FAO-56 Penman [40] models were derived from empirical equations using climate data (e.g., solar and extraterrestrial radiation). Lake ice formation and break-up dates for open lake evaporation, and snowpack measurements for snowmelt runoff modelling were obtained from KazHydroMet (https://www.kazhydromet.kz/). Groundwater level records are not publicly available. Therefore, daily water level records were obtained from the weather station, and used to validate the model and to estimate groundwater flux. Monthly values of water abstracted from the lake between 1989 and 2016 were obtained from the local water agency.

#### Remotely sensed data

A Shuttle Radar Topography Mission Digital Elevation Model (SRTM DEM) with a 30-meter resolution was used to represent the catchment topography. Landsat 5-TM with a 30-meter resolution during the ice-free period and Normalized Difference Water Index (NDWI) were utilised to estimate the lake area in 1986. Similarly, The Normalized Difference Snow Index (NDSI) was used to derive a snow cover area parameter required for the Snowmelt Runoff Model (SRM), which models daily snowmelt runoff.

Leaf Index Area (LAI) required for the calculation of actual evapotranspiration was estimated by MOD15A2H (a MODIS product combining Leaf Area Index and Fraction of Photosynthetically Active Radiation), which is an 8-day composite dataset with 500-meter resolution.

Due to the lack of evapotranspiration measurements, Global Potential Evapotranspiration (Global-PET) geospatial datasets (at 30 arc-second resolution) [41] and Terra MODIS product gap-filled 8-day composite data at 500-meter resolution for potential evapotranspiration (MOD16A3GF PET) were used to validate the evapotranspiration values produced from the catchment. Lake evaporation values were compared with the actual evapotranspiration for the lake using the Operational Simplified Surface Energy Balance (SSEBop) model for the years 2012–2016. Similarly, the outcomes of the snowmelt runoff modelling were compared to monthly surface runoff values produced by the Global Land Data Assimilation System Noah Land Surface Model Version 2.1 (GLDAS) monthly product at 0.25 degree resolution [42] available from 2000 to 2016.

## Methods

### The water balance model

**Fig 2:**
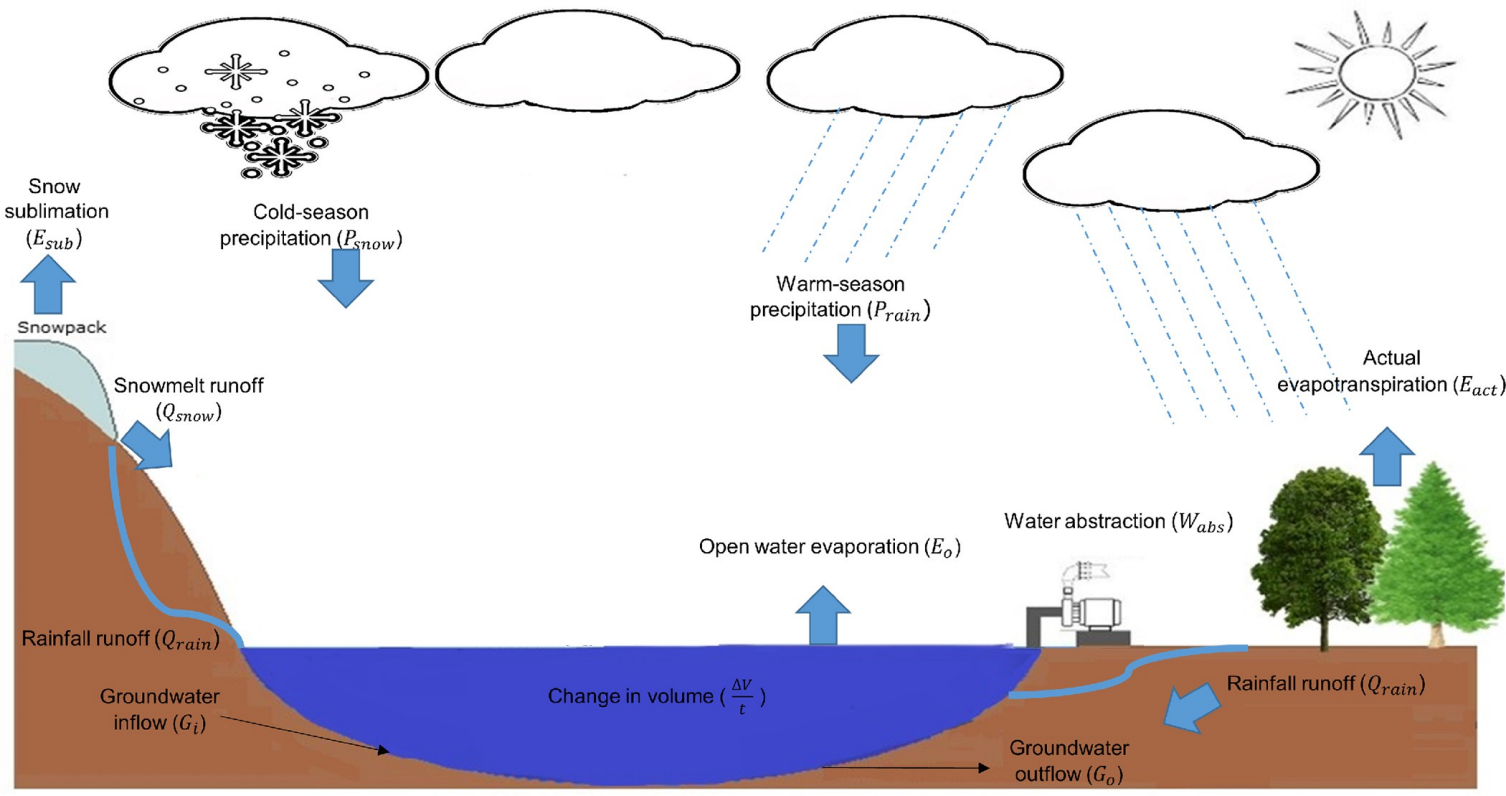
Spatially lumped empirical model for Shortandy Lake.

**Fig 3:**
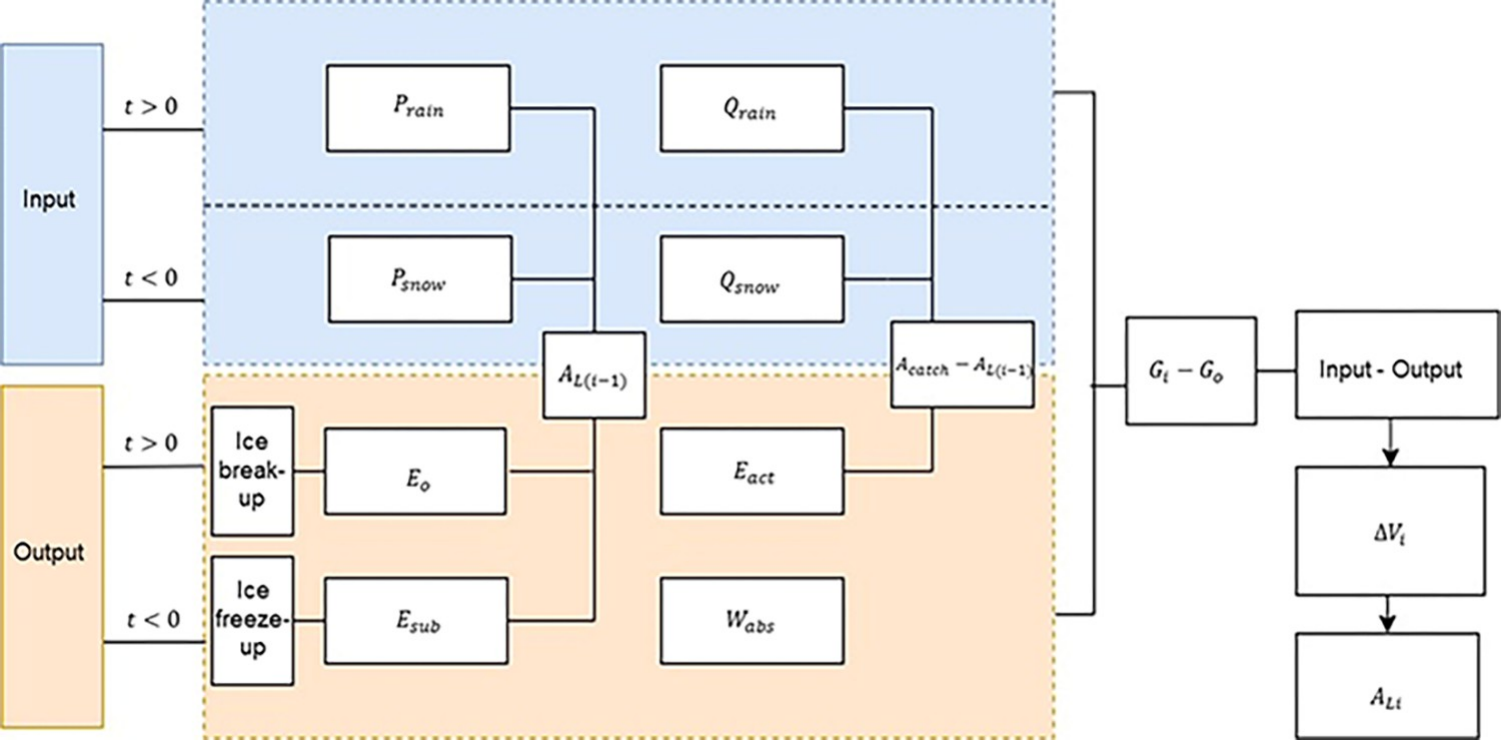
Modelling steps where input and output variables are estimated based on temperature (t) changes. *P*_*rain*_ is rainfall, *P*_*snow*_ is snow, *Q*_*rain*_ is rainfall-generated runoff, *Q*_*snow*_ is snowmelt runoff and *G*_*i*_−*G*_*o*_ is a groundwater flux, *A*_*catch*_ is Shortandy Lake catchment area, *A*_*L(i-1)*_ is the lake area of the previous month, *E*_*o*_ is lake evaporation, *W*_*abs*_ is total water abstraction from surface and groundwater combined, *E*_*act*_ is evapotranspiration from the catchment excluding *E*_*o*_, *E*_*sub*_ is sublimation, *ΔV*_*i*_ is water volume change, *A*_*Li*_ is lake area corresponding to *ΔV*_*i*_, and t is mean air temperature.

A monthly spatially lumped empirical model was developed (Fig 2), defining the water balance as:

$$\Delta V/t=P_{snow/rain}+Q_{rain/snow}-E_{sub}-E_{O}-E_{act}-W_{abs}+(G_{i}-G_{O})$$ (1)

where *V* (m^3^) is volume, *t* is time (month), with inputs of precipitation *P* (mm month^-1^) from snow (*t*<0) *P*_*snow*_ and rainfall (*t*>0) *P*_*rain*_, and runoff from rain *Q*_*rain*_ and snowmelt *Q*_*snow*_ (mm month^-1^). Output variables were sublimation from snow *E*_*sub*_ (mm month^-1^), lake evaporation *E*_*O*_ (mm month^-1^), actual evapotranspiration from the catchment *E*_*act*_ (mm month^-1^), and total water abstraction *W*_*abs*_ (mm month^-1^) from surface and groundwater combined. *G*_*i*_−*G*_*o*_ (mm month^-1^) is the flux of water through subsurface flow delivered to and leaked from the lake. Detailed explanations of the areas associated with each variable required to estimate the volumetric equivalent are provided in Fig 3.

The modelling routine was developed in accordance with the steps reflected in Fig 3. Monthly values of *P*_*rain*_, *P*_*snow*_ *E*_*o*_ and *E*_*sub*_ are a function of the lake area (*A*_*L*_), whereas *Q*_*rain*_, *Q*_*snow*_ and *E*_*act*_ variables depend on the lake catchment area (*A*_*catch*_) without *A*_*L*_. The total amount of water abstraction from groundwater and surface water combined was obtained from the regional water agency—Su Arnasy (https://www.astanasu.kz/). The model results were calibrated between 1986 and 2016, then validated using the lake volume dynamics derived from measured water levels between 2003 and 2016.

### Quantifying change in storage

The relationship between volume and lake area (the final step in Fig 3) were computed using a GIS-based volumetric model. The model was built by merging the SRTM DEM with the bathymetry data (S1b Fig), and interpolated with the Inverse Distance Weighted (IDW) method (S1a Fig). The lake volume and lake area were modelled as a 4^th^-order polynomial in water volume (August 2016), with a significant correlation between the lake area and volume (r^2^ = 0.98, p<0.0001) (S1c Fig). Owing to the lake’s bathymetry, the relationship between lake area and volume is non-linear, where *A*_*L*_ greater than 15km^2^ corresponds to shallow areas of the lake becoming water-filled. The *A*_*L*_ and lake level relationship (S1D Fig) shows that a lake level change occurring between 388–375.5m results in a small change in volume, yet considerable change in *A*_*L*_.

### Lake evaporation

Lake evaporation was estimated for the period when the lake is free from ice cover, using a simplified version of the classic Penman equation [43] developed by Valiantzas [44]:

$$E_{O}\approx 0.051(1-\alpha )R_{S}\sqrt{T}+9.5-2.4{\left(R_{S}/R_{A}\right)}^{2}+0.048(T+20)(1-RH/100)(a_{u}-0.38+0.54u)$$ (2)

where *E*_*O*_ (mm day^-1^) is open water evaporation, *α* is surface albedo for open water (0.08) [45], *R*_*S*_ (MJ m^2^ day^-1^) is solar radiation, *RH* (%) is relative humidity, *u* (m s^-1^) is wind speed, *a*_*u*_ is the wind function for the original Penman equation and is equal to 1, *R*_*A*_ (MJ m^-2^ day^-1^) is extraterrestrial radiation and *T* (°C) is temperature estimated as follows:

$$T=(T_{max}+T_{min})/2$$ (3)

where *T*_*max*_ and *T*_*min*_ (°C) are daily maximum and minimum temperature respectively. *R*_*n*_ for Shortandy Lake was derived from daily solar radiation (*R*_*S*_) and the reflection coefficient (*α*). The wind function was estimated by Linacre [46], where $f_{u}^{\left(3\right)}=0.54u$ for larger lakes (greater 10km^2^).

### Runoff model

The Snowmelt Runoff Model (SRM) [47] was used to evaluate daily *Q*_*snow*_ from the catchment. The SRM and model parameters are described in detail in Martinec [47]. The SRM is a semi-distributed hydrological model that simulates daily catchment runoff and it can also forecast snowmelt. Despite limited climate and hydrological data for Shortandy Lake, SRM estimates surface runoff with minimal input parameters. Daily *Q*_*snow*_ in the Shortandy catchment was calculated using SRM, as:

$$Q_{n+1}=[c_{Sn}*\propto_{n}(T_{n}+{\Delta T}_{n})*SCA]*A*0.116*(1-k_{n+1})+(Q_{n}k_{n+1})$$ (4)

and adding the rainfall-generated runoff:

$$+[c_{rn}{*P}_{n}]*A*0.116*(1-k_{n+1})+(Q_{n}k_{n+1})$$

where *Q*_*n*+1_ (m^3^ s^-1^) is the average daily discharge, *c* is the runoff coefficient expressing the losses as a ratio (runoff/precipitation) with *c*_*Sn*_ referring to snowmelt *c*_*rn*_ to rainfall, *α*_*n*_ (cm °C^-1^ day^-1^) is the degree-day factor indicating the snowmelt depth from one degree-day, *T*_*n*_ (°C day) is the number of degree-days above the base of 0°C, Δ*T*_*n*_ is the adjustment by temperature lapse rate; *SCA* is ratio of the snow-covered area of the catchment; *P*_*n*_ (cm) is precipitation on *n* day contributing to runoff, *A* (km^2^) is area of the catchment, *k*_*n*+1_ is the recession coefficient indicating the decline of discharge in a period without snowmelt, *n* is the number of degree days (°C d) and 0.116 is the conversion factor from cm km^2^ day^-1^ to m^3^ s^-1^.

The elevation range of the catchment is below 500m, meaning the extrapolation of temperature with elevation due to lapse rate is not necessary (Fig 1). The SRM model requires a degree-day factor that considers snow density properties for all elevation zones, regardless of land cover types. However, snow density changes throughout the melt season and is influenced by both land cover type and snow albedo [48]. Thus, Eq 4 was re-written as follows:

$$Q_{n+1}=c_{SnF}[\propto_{Fn}(T_{n}+{\Delta T}_{n}){SCA}_{F}]*A_{F}*0.116+c_{SnG}[\propto_{Gn}(T_{n}+{\Delta T}_{n}){SCA}_{G}]*A_{G}*0.116*(1-k_{n+1})+Q_{n}k_{n+1}$$ (5)

where *F* and *G* refer to forest and grassland respectively. The empirical degree-day factor was updated according to Kuusisto [49] equations, where degree-day was $\alpha_{Fn}=10.4\frac{\rho_{Fs}}{\rho_{w}}-0.7$ for forest and $\alpha_{Gn}=19.6*\frac{\rho_{Gs}}{\rho_{w}}-2.39$ for grassland, where *ρ*_*Fs*_, *ρ*_*Gs*_ (kg m^3^) is the snowpack density in forest and grassland respectively; forest and grassland areas are derived based on land cover maps (S2 Fig).

Snow-cover area (SCA) is another important input to the SRM. The NDSI for grasslands and the relationship between point-measured snow water equivalent (SWE) and degree-day factor for forests were utilised to simulate the SCA. Depletion of snow in forest areas was estimated as a linear function of SWE and degree-day factor (snowmelt depth) which results in an SCA reduction the following day. The recession coefficient (*k*) was within the range of 0.2–1.0, where the lowest coefficient is established at the start of the melt season and increases towards 1.0 at the end.

In the SRM, runoff coefficients (*c*_*Sn*_, *c*_*rn*_), which are applied to *Q*_*rain*/*snow*_, can be adjusted at half-monthly intervals to accommodate seasonal variations in evapotranspiration or, more broadly, to address systematic under- or over-estimations of simulated runoff volumes. Losses of *Q*_*snow*_ at the beginning of the snow-melt period were assumed to be minor, due to the minimal effect of evaporation from the snow-covered surface, and *c*_*Sn*_ is near 1.0 [44]. After that, when the growing season starts, more losses must be expected due to evapotranspiration and interception, causing the runoff coefficient to decline. Thus, the *c*_*Sn*_ value ranged from approximately 1.0 to 0.5. The *c*_*rn*_ value, which changes based on evapotranspiration losses during the warm season was replaced by estimated *E*_*act*_.

The outputs of the SRM were evaluated by calculating the Nash-Sutcliffe coefficient (*R*^2^) and also the volume difference (*D*_*V*_) to understand the SRM’s performance. These metrics were estimated as follows:

$$R^{2}=1-\frac{\sum_{i=1}^{n}{\left(Q_{i}-Q_{i}^{\prime }\right)}^{2}}{\sum_{i=1}^{n}{\left(Q_{i}-\bar{Q}\right)}^{2}}$$ (6)

where *Q*_*i*_ is measured monthly discharge, (m^3^ s^-1^), $Q_{i}^{\prime }$ is computed monthly discharge, (m^3^ s^-1^) and, $\bar{Q}$ is the average measured monthly discharge (m^3^ s^-1^).

$$D_{V}=\frac{V_{R}-V_{R}^{\prime }}{V_{R}}*100$$ (7)

where *D*_*v*_ is deviation of the runoff volume, %, *V*_*R*_ is measured seasonal runoff volume and $V_{R}^{\prime }$ is computed seasonal runoff volume. Positive values of *D*_*v*_ indicate that the SRM underestimates seasonal runoff values, whereas negative shows overestimation. Due to the absence of discharge measurements in this area, monthly discharge data from Severny station (53°68′N, 69°63′E; GRCD station code 2311340) were obtained from The Global Runoff Data Centre (GRDC) (https://portal.grdc.bafg.de).

### Groundwater flux

Two methods were used to estimate groundwater flux: (i) using observed water levels and (ii) using the water balance approach. Both model outcomes were compared, and then validated with observed lake levels.

#### (i) Groundwater flux estimated by measured water levels

This approach estimates groundwater flux when the lake surface is fully covered with ice, and was introduced by Uryvayev [32]. A water balance for the cold season gives a better understanding of groundwater interactions within BNNP lakes and can be accurately estimated in these lakes. Based on this approach, the groundwater flux of Shortandy Lake can be evaluated as:

$$G_{i}-G_{o}\approx V_{b.w}-V_{e.w.}+h_{s}-\sum_{cold}W_{abs}$$ (8)

where *V*_*b*.*w*_ and *V*_*e*.*w*._ (m^3^) are lake volume before and at the end of winter respectively, *h*_*s*_ (m^3^) is water content from snowpack formed on the lake surface by the end of winter, and ∑_*cold*_*W*_*abs*_ (m^3^) is the water abstracted during the cold season. The assumption is based on the relationship between *G*_*i*_ and *G*_*o*_ being constant during the year.

#### (ii) Water balance approach

This estimates the groundwater flux by solving Eq 1 where the annual groundwater flux was expressed as:

$$\left(G_{i}-G_{O})=(V_{begin}-V_{end})-(\sum P+\sum R_{\frac{rain}{snow}}-\sum E_{sub}-\sum E_{O}-\sum E_{act}-\sum W_{abs}\right)$$ (9)

where (*V*_*begin*_−*V*_*end*_) (m^3^) is the annual water volume changes estimated from water levels, ∑*P* (m^3^) is annual precipitation falling on the lake surface, $\sum R_{\frac{rain}{snow}}$ (m^3^) is annual runoff from the lake catchment, ∑*E*_*sub*_ (m^3^) is annual snow sublimation from the lake surface, ∑*E*_*O*_ (m^3^) is annual open lake evaporation, ∑*E*_*act*_ (m^3^) is annual actual evapotranspiration, and ∑*W*_*abs*_ (m^3^) is annual water abstraction.

### Model input and parameterisation

The model input includes snow sublimation, potential evapotranspiration and land cover maps. The actual evapotranspiration (*E*_*act*_) was critical to the simulation of effective runoff which contributes to the lake volume after excessive rainfall (when *P*_*rain*_>*E*_*act*_). *E*_*act*_ was calculated from the potential evapotranspiration (PET) using FAO-56 Penman [40] and crop coefficient:

$$k_{c}:\;E_{act}=E_{pet}*k_{c}$$ (10)

where *k*_*c*_ crop coefficient. The ratio was estimated as follows:

$$k_{c}=k_{c,min}+(k_{c,max}-k_{c,min})(1-e^{-0.7LAI})$$ (11)

where *k*_*c*,*min*_ *k*_*c*,*max*_ are the minimum and maximum crop coefficient for deciduous forest during the mid-season (July) respectively, and LAI is leaf area index. In this study, *k*_*c*,*min*_ is equivalent to 0.9, as recommended for deciduous forest [40], *k*_*c*,*max*_ was adjusted for semi-arid climates as suggested by Allen (1998):

$$k_{c,max}=k_{c,min}+[0.04(u_{2}-2)-0.004({RH}_{min}-45)]({h/3)}^{0.3}$$ (12)

where *u*_2_ (m s^-1^) is the wind speed at 2m, *h* (m) is the mean maximum tree height, taken from field observations [50], and was equal to 9m on average.

Snow sublimation (*E*_*sub*_) was estimated to evaluate monthly losses from the ice-covered lake surface during cold seasons. The following parameter was evaluated using correlation analysis between air humidity and snow ablation in Northern Kazakhstan established by Semenov [51]. Hence, losses for snow sublimation were expressed as follows:

$$E_{sub}=n(0.35VPD-0.06)$$ (13)

where n is the number of days in a month and *VPD* (kPa) is the mean monthly vapour pressure deficit. Snow sublimation was used in the *G*_*i*_−*G*_*o*(i)_ model and for snowmelt runoff simulations. Snowpack losses from the lake surface were incorporated in the SCA simulations and thus were excluded to avoid double-counting.

Land cover maps were required to derive grassland and forest areas, as well as the SCA variable in Eq 5 and estimate LAI parameter in Eq 11. The land cover classification assessed changes in i) forest, ii) grassland, and iii) lake areas from 1986 to 2016. Urban areas (less than 5km^2^) were classified as grassland due to the absence of snowpack measurements. Land cover types were evaluated using supervised classification in ArcGIS Spatial Analyst. Analysis showed forest area decreased from 45.7km^2^ (1986–2009) to 39.9km^2^ (2010), while grasslands expanded from 6.7km^2^ to 12.5km^2^ (S2 Fig).

### Sensitivity analysis

Sensitivity analysis of the lake model was performed for different model parameters to assess their influence on the water balance. Four model parameters were considered: (1) air temperature, (2) precipitation, (3) wind speed, and (4) relative humidity, to which simulated lake level estimates are most often attributed to [52, 53], with the last two parameters are embedded in the open water evaporation (Eq 2). Based on the variation of the selected parameters within the sensitivity analysis, groundwater flux was also evaluated, as these parameters ultimately affect groundwater flux. Sensitivity analysis was conducted following a ‘one at a time approach’ and using the Sensitivity Index [54], as follows:

$$I=\frac{(y_{2}-y_{1})/y_{0}}{2\Delta x/x_{0}}$$ (14)

where *I* is the sensitivity index, *y*_0_ was the initial model output estimated with an initial *x*_0_ of the parameter *x*. In the sensitivity analysis, this initial parameter value varied by 10–25%, with a 5% increments while others were kept constant, yielding:

$$x_{1}=x_{0}-\Delta x,$$

$$x_{2}=x_{0}+\Delta x$$ (15)

where corresponding values *y*_1_ and *y*_2_ were the lake storage. Changes in the lake storage based on the sensitivity analysis were then compared to the ‘initial model’, which is the lake storage estimations calculated from Eq 1.

## Results

### Model validation and sensitivity

**Fig 4:**
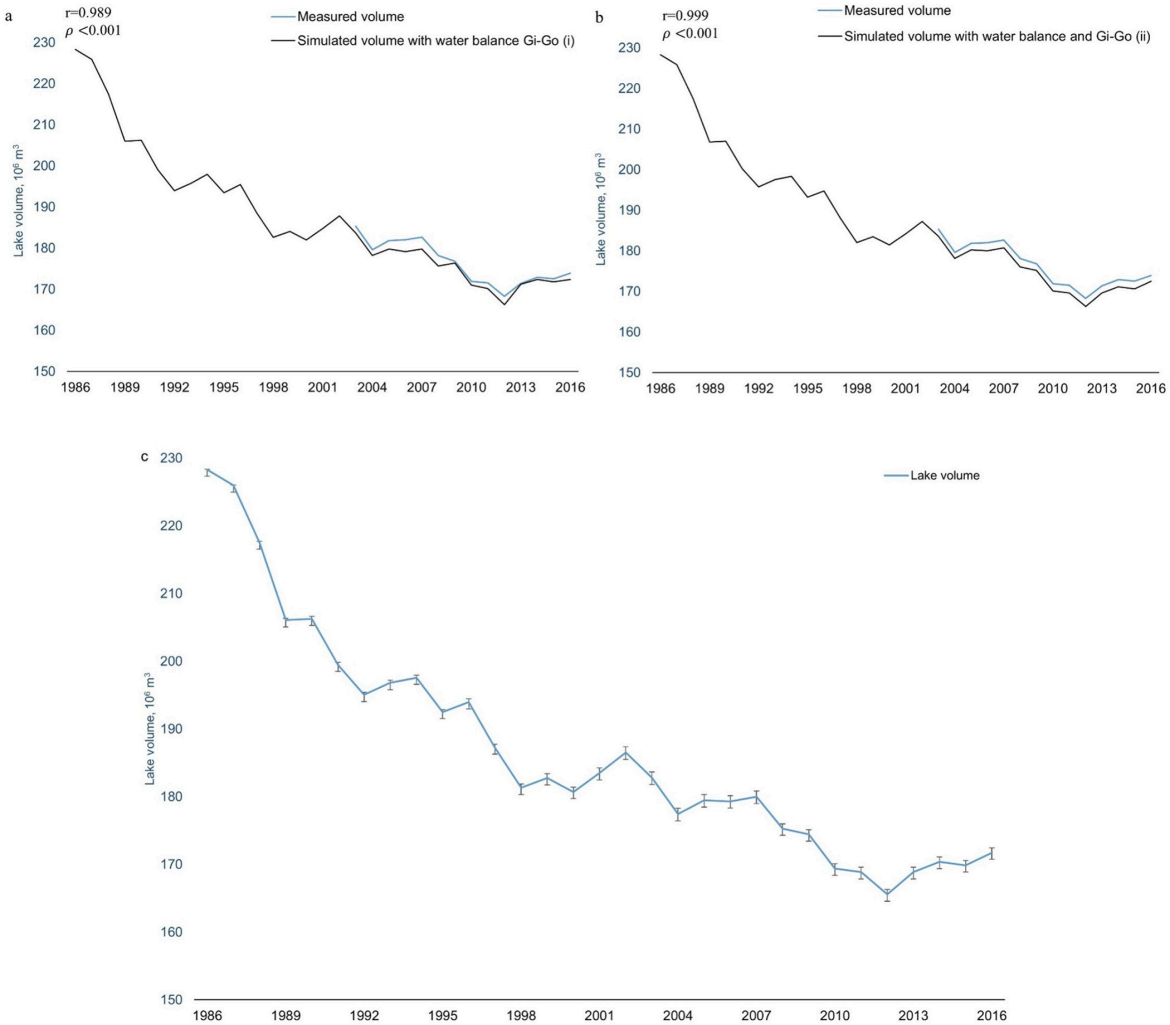
Model validation for Shortandy Lake. (a) volume simulated by the regional groundwater model, (b) volume simulated by groundwater flux estimated by water balance approach, and (c) water volume dynamics estimated by Eq 1, where errors bars show standard deviation of the lake volume obtained from a one-by-one sensitivity analysis with four selected model parameters.

The observed lake volume and the volumes simulated by Eq 1 with two groundwater flux models were both statistically significantly correlated (r = 0.999, *p*<0.001 and r = 0.989, *p*<0.001) (Fig 4A and 4B). The difference between the simulated lake storage and volume estimated by water levels, was below 10% of the total lake volume. Although the lake storage simulated using groundwater model (ii) showed a higher correlation with measured lake volumes, groundwater model (i) showed minimal deviation between simulated and observed lake storage, especially for wet years (2009–2010 and 2013–2014).

The sensitivity analysis indicates that the model shows negligible sensitivity when model parameters are varied within the range of 10%, and the model could reproduce adequately the lake storage changes (r = 0.99, *p*<0.001). However, the model was sensitive to precipitation when variation exceeded 10%. The sensitivity analysis indicated that the lake volume is highly responsive to variability in precipitation, particularly during wet years, which affect runoff values. In contrast, the model showed less sensitivity to air temperature, and even less sensitivity to wind and relative humidity.

Sensitivity analysis on the groundwater flux changes indicated minor variability owing to changes in wind speed. Both relative humidity and temperature had a similar effect on *G*_*i*_−*G*_*o*_. In contrast, precipitation had the most significant impact on groundwater flux, especially during wet years, emphasizing the model’s sensitivity to precipitation.

The model sensitivity increases over time (Fig 4C). The overall model accuracy is affected by the lack of lake level observations for 1988–1990 and 1998–2002, which are required for the estimation of *G*_*i*_−*G*_*o*_. Consequently, these factors affect the accuracy of the model during the aforementioned periods.

### Reconstruction of water volume dynamics

The modelling outcomes show a significant water volume reduction in Shortandy Lake (r = -0.93, *p*<0.001), from 231.7x10^6^m^3^ in 1986 to 172.5x10^6^m^3^ by the end of 2016 (Fig 4). The most rapid and dramatic water volume decrease occurred between 1986 and 1992. Despite inter-annual water volume fluctuations, with positive water volume dynamics (1992–1996; 2002; and 2005–2009), the overall trend was downward. However, since 2013, the water volume trend became positive after the lowest values simulated in 2012 (≈166.3x10^6^m^3^), rising to 172.5x10^6^m^3^ by 2016.

### Historical changes in output variables

#### Open lake evaporation

**Fig 5:**
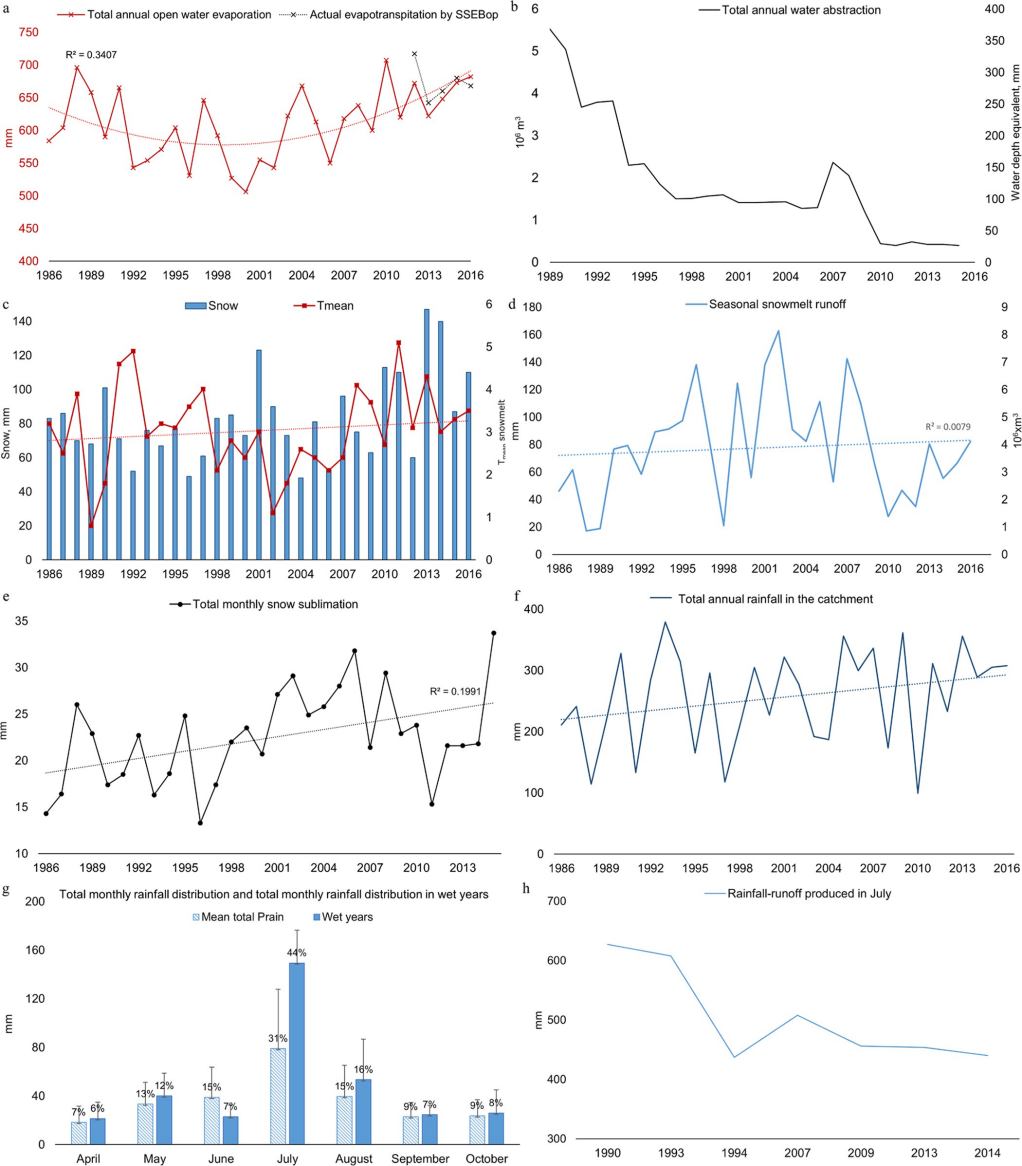
Historical changes water balance variables of Shortandy Lake. (a) Total annual open water evaporation estimated by the simplified Penman equation and actual evapotranspiration by SSEBop is the actual evapotranspiration for the lake by Operational Simplified Surface Energy Balance model, (b) Total annual surface and groundwater abstraction, (c) Total annual snow (*P*_*snow*_) and mean temperature during snowmelt, (d) Total seasonal snowmelt runoff, (e) Total annual snow sublimation, (f) Total annual rainfall in the catchment, (g) Total monthly rainfall distribution and total monthly rainfall distribution in wet years (excessive rainfall events), and error bars show a standard deviation, (h) Total monthly rainfall-runoff produced in the catchment.

The temporal evaporation trend showed no significant change (r = 0.3, *p* = 0.08), with an average of 611mm year^-1^. Estimations of lake evaporation flux revealed two periods with different lake evaporation patterns (Fig 5A). Between 1986–2005 lake evaporation flux remained stable (r = 0.04, *p* = 0.37) followed by a slight increase between 2006–2016 (r = 0.4, *p*<0.05). Annual *E*_*o*_ increased on average from 594mm (1986–2005) to 682mm per year (2006–2016), with a maximum value of 707mm in 2010. The ice break-up dates, used to identify open lake evaporation periods revealed a significant, strong negative relationship between the lake ice-off dates and the air temperature deviation of April and March (r = -0.78 and *p<*0.001) (S3C Fig). Lake ice-off showed a weak negative temporal trend (r = -0.47, *p*<0.05), where ice-free conditions were longer since 2005.

#### Water abstraction

The total water volume abstracted from the lake during the study period (1986–2016) was 51.4x10^6^m^3^, equivalent to 2.3x10^6^m^3^ of water annually (Fig 5B). The greatest proportion of water was abstracted between 1989 and 1993, with the maximum annual abstraction in 1989. Since 1996, water abstraction from Shortandy was approximately 1.6x10^6^m^3^ annually, until the government instated a policy to reduce water abstraction from the lake to around 0.5 million m^3^ annually in 2010.

### Historical changes in input variables

#### Snowmelt runoff

The snowmelt season was highly variable in terms of both the snowpack and its distribution across the catchment, with temperature controlling the duration of the snowmelt season. The estimated parameters used for the model are shown in S3 Fig. Temporal correlation analysis indicates a significant but weak positive trend in snow, (r = 0.41, *p* = 0.02) (Fig 5C). Snow values reached their maximum values in 2013 and 2014, however the highest *Q*_*snow*_ values were observed in 2002 and 2001 (Fig 5D). This can be attributed to the increased temperature during the snowmelt season since 2010 (Fig 5C), which increased water losses from evapotranspiration. Furthermore, the total amount of *P*_*snow*_ and SWE accumulated during the cold-seasons (S3D Fig) is likely to be influenced by increased losses from snow sublimation since 2000 (Fig 5E), which, in turn, impacted seasonal *Q*_*snow*_.

The overall trend shows a significant reduction in snowmelt duration by 12 days (r = 0.5, *p<*0.001) (S3E Fig). Snowmelt duration in the forested area of the catchment was driven by *T*_*mean*_ and SWE, where the snowmelt duration shows a significant moderate negative relationship between *T*_*mean*_ (r = -0.6, *p*<0.001) and a significant (but weak) positive relationship with SWE (r = 0.4, *p*<0.05). The relationship between snowmelt duration in grassland was statistically significant with the air temperature only (r = 0.61, *p*<0.001).

Seasonal snowmelt runoff remained unchanged during the study period (r = 0.1, *p* = 0.5), with, on average, 71mm of water contributing to the lake volume annually (Fig 5D). *Q*_*snow*_ in the catchment varied inter-annually; the lowest runoff was simulated in 1988, 1989 and 1998 (less than 20mm of the lake depth), while the highest peak of runoff was in 2002 and 2001 (more than 130mm).

#### Rainfall-runoff

There is interannual variability in rainfall, with the average annual total rainfall value of 256 mm year^-1^ with a standard deviation of 80mm over the study period. Temporal correlation indicates no significant change in rainfall during the study period (r = 0.26, *p* = 0.17) (Fig 5F). Fig 5G compares the monthly distribution of the total mean rainfall with wet years, during which the highest values of rainfall occurred in July. The average was 31% (79mm) out of the annual rainfall, while in wet years, July rainfall comprised 44% (149mm) of the annual total. The lowest amount of rainfall occurred in April (7–6%) and the autumn months with 9–7% of the total annual rainfall. The SRM simulations showed high variability in *Q*_*rain*_, with rainfall values exceeding *E*_*act*_ only in July (Fig 5H). For example, in 1990 and 1993 runoff from rainfall was greater than 600mm month^-1^; however, after the lowest *Q*_*rain*_ value estimated in 1994, the runoff remained below 450mm.

*E*_*act*_, necessary for assessing *Q*_*rain*_, was estimated using FAO-56 Penman potential evapotranspiration (PET) and crop coefficient.The crop coefficient was determined for a forested area of the catchment, with *k*_*c*_ ranging from 0.8 to 0.9. The total annual *E*_*act*_ was on average 625mm, and 129mm in July with a standard deviation of 22mm (S3B Fig).

### Groundwater flux

The groundwater flux was estimated using two different approaches (S4 and S5 Figs). The groundwater modelling showed that groundwater flux was positive, but the groundwater recharge was small. The average *G*_*i*_−*G*_*o*_ was +0.29x10^6^m^3^ when estimated by the water level approach (i) and +0.16x10^6^m^3^ when estimated by the water balance approach (ii) (S4 and S5 Figs). Groundwater predictions from both models showed similar trends (r = 0.84, *p*<0.001), especially between 2003 and 2011, illustrating similar patterns in groundwater flux.

**Fig 6:**
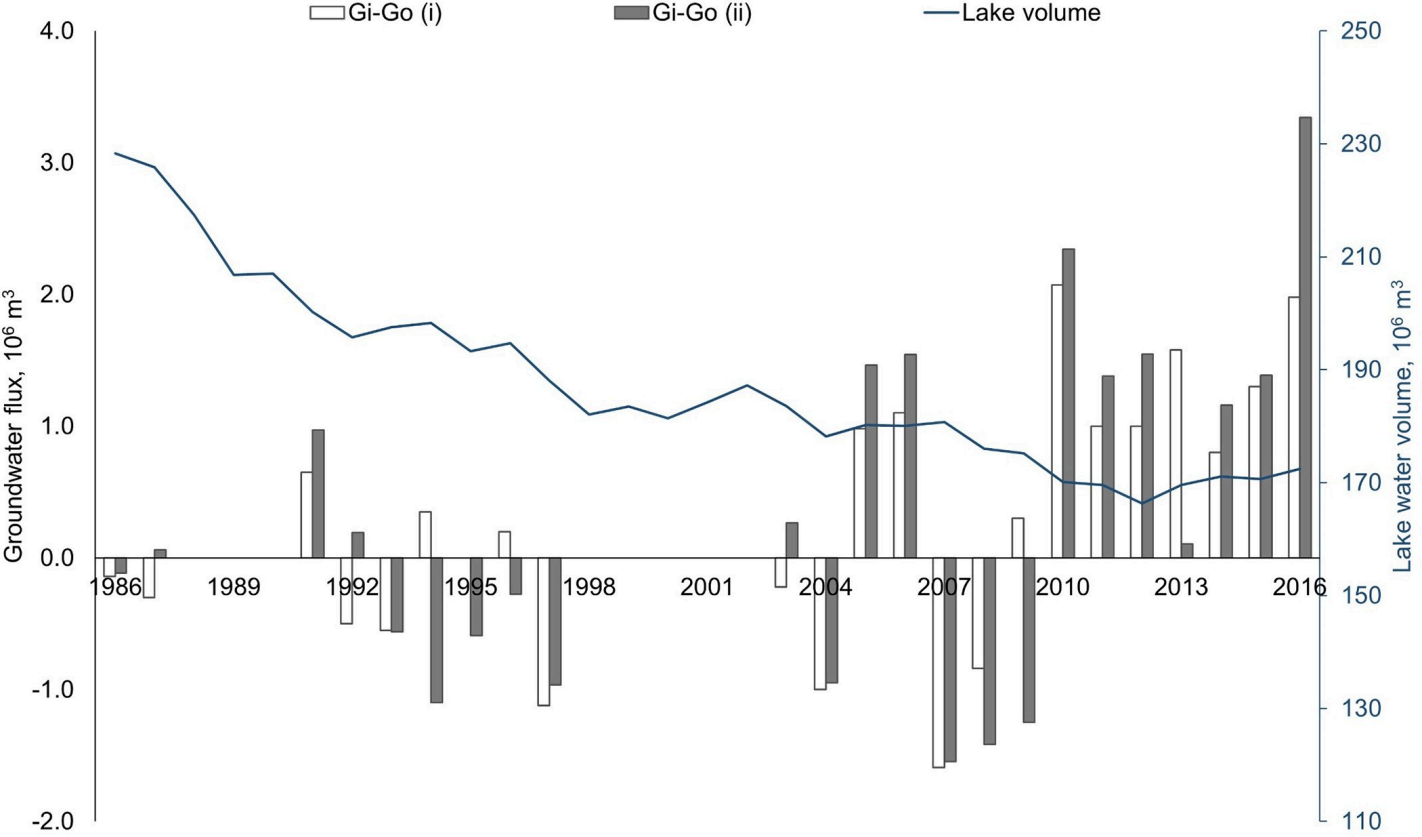
Groundwater flux modelling. *G*_*i*_−*G*_*o*_ (i) is groundwater flux estimated using the measured water level approach; *G*_*i*_−*G*_*o*_. (ii) is estimated using the Shortandy water balance model in Eq 1.

The results showed a clear relationship between Shortandy Lake volume and groundwater flux (Fig 6). When the lake volume was high (during the 1980s-1990s), the groundwater flux was negative. A positive response in groundwater flux with considerable inflow was established from 2010 onwards, when the lake volume reached the lowest level during the study period. This relationship was also reported by Uryvayev (1958) [32], who stated that significant groundwater recharge stabilises water level fluctuations by recharging lakes when they reach their minimal water level.

Major discrepancies between the two groundwater modelling approaches were observed for 1994 and 2013 (Fig 6). Specifically, in 2013, there was a discrepancy of one million m^3^ of water between the two modelling approaches. The deviation in *G*_*i*_−*G*_*o*_(ii) could result from an underestimation of groundwater inflow during warm season months. Specifically, the highest discrepancy corresponds with wet years when annual *P*_*rain*_ is more than 350mm.

### Changes in net flux

**Fig 7:**
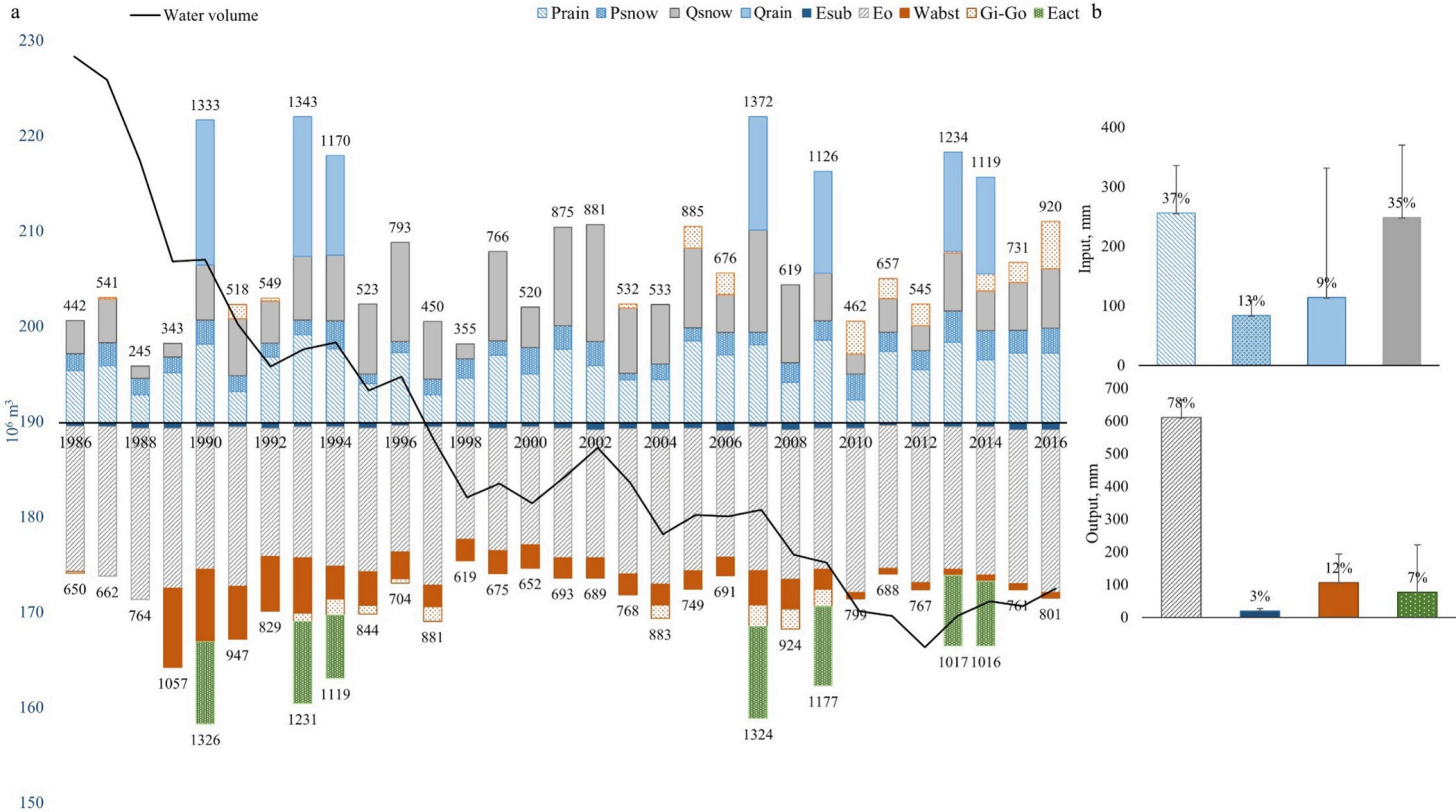
Water balance model outcomes for Shortandy Lake. (a) Total annual input and output variables (mm) (b) Relative contribution and mean values of input and output variables from 1986 to 2016, where error bars show standard deviation.

The inflow-outflow water balance of Shortandy Lake was predominately negative during the study period, and the net balance was -95mm (Fig 7A). A series of dry years occurred between 1986–1992. During these years, there was a decrease in annual *P* and a reduction in snowmelt runoff (except 1990). By contrast, the total annual output increased considerably due to the water abstraction from the lake, with the relative contribution to the output reaching more than 25%.

During wet years, the positive water volume response was on average equal to 88mm where the highest positive net balance (250mm) was estimated for 2013. Wet years were driven by increases in rainfall of around 30% above the long-term average. However, an increase in the input variables for years with extensive summer rainfall events made little contribution to the lake volume due to losses from evapotranspiration. Since 2013, wet years made a greater contribution to the lake volume. Although the evapotranspiration proportion was higher in 2013–2014 than during the 1990s (33%-30% and 29% respectively), the proportion of the anthropogenic water abstraction reduced from 20% to 4%, which in turn resulted in a positive water volume response.

Overall, dry periods repeated on average every 3–4 years, followed by 1–2 wet years, over the study period. The input variables showed inter-annual variation, where the highest deviation was established for *Q*_*snow*_ and *Q*_*rain*_ (Fig 7B). In dry years total precipitation declined by 30%, whereas the proportion of snowmelt runoff varied considerably (34%-17% of annual input) with increased losses from lake evaporation.

### Model evaluation

The lake evaporation model exhibited good performance, with an average annual underestimation of 8mm compared to actual evapotranspiration values for the lake by SSEBop. When comparing the estimated *E*_*o*_ and *E*_*o*_ (SSEBop), both a t-test and Pearson correlation analysis suggested a moderate linear relationship with no statistically significant difference for both annual and monthly values (t-value = 0.3, r = 0.57, *p* = 0.3) (Fig 5A) for the years 2012–2016. Moreover, a strong correlation was found for the monthly average estimated *E*_*o*_ and *E*_*o*_ (SSEBop) (r = 0.95, *p*<0.001) (S3A Fig). The highest deviation between estimated *E*_*o*_ and *E*_*o*_ (SSEBop) occurred when lake evaporation was overestimated for summer months and underestimated for autumn months. This deviation is likely associated with the absence of heat storage capacity in Eq 2. Lakes with an average depth exceeding 15m exhibit a one-month lag between net radiation and evaporation flux [55], causing the model to overestimate evaporation in summer due to increased net radiation and, conversely, to underestimate it during autumn. The assessment of the simplified Penman equation [44] demonstrates that the model performs accurately, not only for Shortandy Lake but also for other semi-arid lakes [56, 57].

Similarly, both satellite-derived evapotranspiration values (Global-PET and MOD16A3GF PET) were compared with evapotranspiration estimated using field-observed data (S3B Fig). A strong correlation was found for all three models (*E*_*act*_, Global-PET, MOD16A3GF PET), where the highest correlation established with monthly MOD16A3GF PET and *E*_*act*_ (r = 0.99, *p*<0.001). *Q*_*rain*_ contributed to the lake volume most in July, with *E*_*act*_ and MOD16A3GF PET average values of 130-136mm, respectively (1990, 1993, 1994, 2007, 2009, 2013 and 2014). The highest deviation between *E*_*act*_ and MOD16A3GF PET was established during the spring and autumn seasons. There was an overestimation in April to June (on average 65mm) and nearly equal underestimation between August and October (-67mm), resulting in a negligible annual average difference of -4mm. In the Akmola region, recent studies on potential evapotranspiration from 1991 to 2021 showed a decreasing trend, ranging between 632mm and 850mm, with no significant change observed, particularly in the BNNP area [58].

The assessment of results from the influx model was also critical. According to measurements from Severny station, the majority of streamflow occurred during the snowmelt season, i.e., from March to May, with the peak values in April, which is consistent with the SRM simulations. The GRDC dataset shows that most streamflow measurements ceased in 1987 due to the closure of most stations within the study area. As a result, streamflow data for only two years can be compared with the SRM simulations (1986 and 1987). Using discharge data from the station, *Q*_*snow*_ was calculated for months when snowmelt occurred, resulting in runoff values of 35mm and 69mm, compared to 38mm and 50mm simulated by the SRM in 1986 and 1987, respectively. The accuracy assessment of SRM outputs showed an average Nash-Sutcliffe determination coefficient (*R*^2^) of 0.98 and the mean absolute value of the volume difference (*D*_*v*_) of 5.1%.

Additionally, *Q*_*snow*_ was compared to monthly surface runoff values produced by GLDAS for 2000–2016. The analysis indicates a positive but weak and statistically non-significant linear relationship between GLDAS and SRM (r = 0.36, *p* = 0.15) (S3F Fig). The performance of the SRM can be considered fair, as the difference between the SRM and GLDAS was on average 24mm. The highest difference occurred between 2013 and 2016, coinciding with years of maximum snowfall (except for 2016). In another study, similar findings were observed in Chinese basins, where a substantial increase in errors was noted within the GLDAS dataset during the transition from precipitation to runoff data [59]. The average annual *Q*_*snow*_ was 71mm and 105mm according to SRM and GLDAS, respectively, with SRM runoff corresponding to the global average annual surface runoff produced for Shortandy by Fekete [60].

There are numerous runoff models that exist, ranging from empirical to physically-based approaches, where the choice of the model is determined by application objectives and available input data. Both empirical and conceptual models have been favoured for use in the CA region [61–64] due to the lack of gauging networks and data quality. In snow-dominated areas with established long-term runoff measurements, employing statistical methods provides an alternative approach for runoff estimation [65, 66]. Conceptual snowmelt models, e.g., SRM, also referred to as the degree-day factor model, have been successfully applied in snow-fed catchments for accurate runoff modelling [67–70]. Despite having a simple model structure, the degree-day method has comparable accuracy to process-based models [64], achieved through improvements in the degree-day factor and modifications to the model structure [71, 72], as supported by several studies [67, 73, 74]. For example, a comparative analysis between SRM and the semi-distributed process-based variable infiltration capacity model (VIC) showed a difference of around 10% in simulated results for the Upper Indus Basin [67].

While uncertainties in SRM simulations are widespread for high-elevation and glacial regions [75], for low and mid-elevation areas, the model’s most sensitive variables are the degree-day factor and snow cover area [68]. In this study, the original degree-day factor was refined based on the vegetation coverage using land cover maps (S2 Fig), and was calibrated using recession coefficients. The high determination coefficient of the SRM for Shortandy Lake indicates that this model provides robust results for forested catchments. This finding aligns with previous studies [73, 76, 77], where the degree-day factor model exhibits better performance in forested areas. This is attributed to temperature being a major indicator of the surface energy balance in forested areas, where the canopy mitigates the effects of direct solar radiation and wind [78].

Eq 1 requires the accurate estimation of the lake area, with variables such as *P*_*rain*_, *P*_*snow*_ and *E*_*o*_ being functions of the monthly lake area. The lake area-volume-level relationship is sensitive to changes occurring when *A*_*L*_ exceeds 15km^2^ or when the lake level is above 388m, corresponding to the shallowest areas of the lake (S1D Fig). Therefore, in this range, any variations in volume result in a considerable change in *A*_*L*_. However, a substantial change in *A*_*L*_ is likely to occur when the lake level drops below 388m, leading to a reduction from 16.3 to 14.6km^2^, with a relatively small change in water volume (around 7x10^6^ m^3^). It is worth noting that this study did not observe the water level reaching this specific threshold, however.

## Discussion

### Impacts of water abstraction on changes in water storage

**Fig 8:**
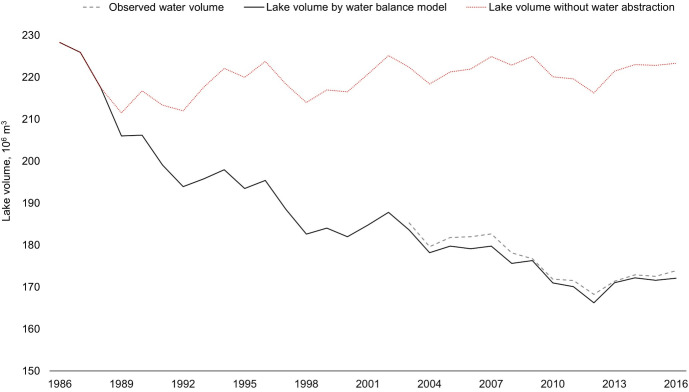
Assessment of the impact of water abstraction on Shortandy Lake. The grey line shows water volume estimated by measured lake levels, the black line is the lake volume simulated by the water balance model, and the red line shows the water volume changes without water abstraction.

To assess the role of anthropogenic impacts on lake volume changes, the *W*_*abs*_ parameter was excluded from Eq 1 and the water balance recomputed. Fig 8 shows that the volume would have been relatively stable and well above the water volume observed when water abstractions are omitted. This suggests that climate change played only a minor role in the water volume decline, with small inter-annual variability. Our findings indicate that the reduction in water volume in Shortandy Lake is primarily caused by anthropogenic water abstraction, which caused 92% of the total water volume deficit (i.e. 59x10^6^m^3^). The highest levels of water abstraction (greater than 25% of the annual outflow) coincided with the driest periods (1988–1989, 1998) of the study period. The negative net water balance caused a significant decline in the lake volume of Shortandy Lake and the lake volume did not naturally recharge during wet periods (1993–1994, 1999–2002, 2007, and 2009). A positive recharge of the lake volume was observed only after 2013 when water abstraction was reduced due to policy changes.

Endorheic lakes and surface waters such as rivers and streams are essential sources of freshwater in CA [20]. During the Soviet Union, water abstraction from the BNNP lakes was restricted to serving nearby settlements and a few resorts within the Park (2,000 visitors annually). Previous studies [32] indicated that the lakes had sufficient freshwater resources for local needs, when taking into account variations between dry and wet years. Following the Soviet Union collapse, the government aimed to develop the area into a major tourist destination, leading to increased pressure on the lakes due to the absence of a centralised water supply.

The reduction in the size of endorheic lakes in arid and semi-arid regions presents significant challenges to the sustainable development of ecosystems. A large body of evidence reveals that the primary cause of lake shrinkage in CA is anthropogenic impact. Huang, Duan [79] studied water storage changes in more than 9,000 lakes across the CA region, observing shrinkage in lakes below 3,500m between 1990 and 2020. These reductions were attributed to human activities (rapid population growth, intensification of agriculture, and rising competition for surface water resources) rather than changes in climate. The shrinking of Balkhash Lake can be attributed to dam construction, creation of the Kapchagay reservoir, and increases in land used for agriculture and irrigation [80]. The primary cause of Ebinur Lake’s shrinkage is the over-expansion of irrigated cropland [81], where an increase in annual inflow by 603x10^6^m^3^ and a reduction in human water consumption by 320x10^6^m^3^ would be needed to restore the lake to 522km^2^. Bosten Lake, the largest inland freshwater lake in China, has undergone drastic changes from 1961 to 2016 [82]. Water balance modelling for Bosten Lake revealed that climate-driven regime shifts contributed to some of the lake level changes, but other factors such as ecological water conveyance, agricultural irrigation, and water consumption also played significant roles.

### Impacts of climate on water storage

Climate change is an important factor that can alter the hydrological regime of lake catchments via precipitation, temperature and evaporation [83, 84]. However, even in remote lakes with no direct human impact, distinguishing the effect of climate change on a lake’s water balance is difficult due to the effects of background climate variability and atmospheric teleconnections such as the North Atlantic Oscillation (NAO) and El Niño-Southern Oscillation (ENSO) [85]. The NAO and ENSO are two important factors that influence the precipitation regime in this region, creating interannual and decadal variations [86, 87]. Our results show that the dry periods established at Shortandy in 1988–1989 and 1998 coincide with negative phases of the West Pacific Oscillation released by ENSO over northern Eurasia [88]. This period resulted in reduced snow and subsequently affected seasonal runoff in Shortandy (Fig 5E).

Changes in lake ice phenology with a changing climate is another important determinant of a lake’s water balance [89]. The winter temperature anomalies over CA and the West Siberian region during the past century were associated with the NAO [7, 88]. The NAO is recognised to have a significant impact on air temperature across the Northern Hemisphere, controlling winter precipitation [89, 90]. Records of the ice-freeze and break-up in the lake show that the ice-free condition of the lake has been extended since 2005 (S3C Fig). Similar changes in lake ice phenology has been reported for other lakes located in the Northern Hemisphere [85]. A decrease in lake ice phenology suggests earlier stratification and an increase in the lake surface water temperature, resulting in higher rates of evaporation [19].

Both NAO and ENSO are multidecadal, which makes it difficult to disentangle them from the effects of climate change [91]. The cycle of wet years that occurred in Shortandy confirms results reported by Chen, Huang [92] and Huang, Chen [93] that a cycle of 2–3 years of variation in precipitation is common for North Kazakhstan. Understanding of the relationship between precipitation patterns and large scale atmospheric and oceanic systems in CA is limited [93–95], specifically in the simulation of ENSO [95]. However, Wang, Song [19] revealed a widespread water loss in the global endorheic system during 2002–2016 that is less influenced by short-term climate variability, suggesting a possible response to longer-term climate conditions and human water management.

Globally, from 1984 to 2015, around 90,000km^2^ of surface water evaporated, whereas 184,000km^2^ of water surfaces were formed [85]. Most of these changes are associated with background climate variability, water abstraction and reservoir filling, rather than climate change [96]. Although the hydrological cycle of endorheic lakes is sensitive to climate change, the actual magnitude of change established in the Shortandy catchment that can be undoubtedly attributed to climate change remains unclear, particularly given the key impact of anthropogenic water abstraction. Ramazanova, Bulai [97] findings confirm that water abstraction from the lakes is one of the major factors in the recent water level decline of BNNP lakes. Karthe, Chalov [25] and Lioubimtseva and Henebry [98] also confirm that changes in regional climate are likely to have lesser influence on water resource availability than ineffective water management and overexploitation of water resources in CA.

The water system in endorheic lakes is evidently fragile worldwide, and the uncertainty surrounding fluctuating water resources is further intensified by global warming. However, the Shortandy case shows that CA endorheic lakes face heightened vulnerability to shallowing due to several factors: sparse monitoring as well as inaccessible data hindering the establishment of hydrological models, which in turn complicates the evaluation of past and future climate fluctuations, as well as the accurate prediction of changes in storage. Without up-to-date models, deriving recommendations for water resource management is challenging and could result in unsustainable use of water resources. In the CA region, water is critical resource, and rapidly increasing freshwater withdrawals imply that the continuation of current water use practices may contribute to growing conflicts with other water users, aggravation of water stress, and disputes with neighboring countries [99]. Although lakes are unique in terms of their formation and functioning [100], the model described here can be used for other CA endorheic lakes to assess long-term climate variability and water abstractions on lake storage. This makes it possible to set abstraction limits during dry years, ensuring the sustainable management of endorheic lakes. Our findings suggest that the future development of tourism in most BNNP lakes should be implemented with caution, as further exploitation of water resources may have negative environmental and economic implications for Northern Kazakhstan.

### Modelling challenges and limitations

Our study highlights that the scarcity of field-based observations remains a significant limitation in hydrological modelling for endorheic lakes. Insufficient data introduces considerable uncertainty in the conceptualisation and construction of regional numerical models. The case of Lake Urmia demonstrates that the absence of adequate data and a reliable model makes it impossible to achieve successful restoration actions by policymakers [23]. Karthe, Abdullaev [101] similarly highlight that the absence of data is one of the major challenges in implementing Integrated Water Resource Management (IWRM) in CA.

A significant emphasis is placed on studying the relationship between groundwater and rivers and streams, but there is limited attention given to the interaction with lakes [102]. This might be associated with missing groundwater data, which is not unique, especially for lakes in CA. The utilisation of physically-based groundwater models (e.g. Modflow, SUTRA) requires observed groundwater levels, accurately assessed hydraulic parameters, and well-defined boundary conditions. The lack of these parameters presents significant challenges for groundwater modelling, leading to increased uncertainties that affect the interaction between surface and groundwater, resulting in notable uncertainties in the total water budget [103]. Many studies use remote sensing data (e.g. Gravity Recovery and Climate Experiment (GRACE) and GLDAS [83, 104]), but field data remains crucial for reliable modelling [105]. Among challenges with remotely sensed data is the low spatial resolution, including the inaccuracy of runoff data which can lead to inconsistencies and uncertainties in the simulation of groundwater storage [106, 107], especially for small-catchments. The absence of comprehensive groundwater data in CA lake studies has necessitated the use of simplified empirical lake models, which have proven advantageous, as seen in cases such as Bosten Lake [82], Issyk-Kul Lake [108], the Caspian Sea [109], and Urmia Lake [110].

The sensitivity analysis demonstrated that the inaccessibility of groundwater levels for Shortandy Lake affected the model’s accuracy, resulting in changes to the accuracy of long-term storage simulations. Nevertheless, the approach developed in this study is deemed acceptable for addressing the lack of groundwater data. We successfully modelled all remaining fluxes (e.g., snowmelt and rainfall runoff, lake evaporation, and water abstraction) and then validated using measured lake levels. Despite the unavailability of groundwater data, we employed two different methods to evaluate groundwater storage. The simulated lake storage using both groundwater flux models exhibited statistically significant correlations with the observed lake storage and showed minimal differences in the temporal water balance between the two approaches (Fig 4). Our results also offer valuable insights into the relationship between lake and groundwater storage, providing answers to the question of when groundwater storage becomes significant for lake storage. Specifically, our findings revealed that the assessment of groundwater storage becomes crucial when the volume of Shortandy Lake declines to its minimal threshold (170x10^6^m^3^), as observed in 2012.

The interaction of surface and groundwater in BNNP lakes is still not well understood. Yapiyev, Skrzypek [15] conducted a one-year isotopic analysis for these lakes, suggesting a larger input of groundwater for Shortandy and Burabay. However, continuous monitoring and analysis over an extended period is necessary to capture seasonal variations, interannual variability, and long-term water abstractions for an accurate assessment of the long-term water balance.

The sensitivity analysis showed that Shortandy Lake is highly responsive to variations in precipitation, particularly in wet years, which affects runoff and groundwater fluxes. In this study, precipitation data was based on one station record and is therefore a source of uncertainty due to the small number of rain gauges. Therefore, establishing monitoring stations for various parameters, such as surface runoff, meteorological conditions, and groundwater levels, is critical in water resource assessment. Moreover, data availability is essential for effective surface and groundwater management.

## Conclusions

We developed a spatially lumped empirical model for the data-sparse endorheic Shortandy Lake, which is robust for assessing the long-term impacts of climate variability and water abstractions on lake volume. The model simulations of lake storage were validated and showed a high correlation with measured lake levels, with an error within 10% of the total lake volume. This analysis emphasises the significance and potential advantages of the model, especially when dealing with limited observational data with which to calibrate the model. The model simulations showed that a significant decline in the water volume between 1986–2016 has been predominantly driven by anthropogenic water abstraction. The case of Shortandy Lake here, suggests that the reduction in water storage in endorheic lakes in СA is partially attributed to a lack of up-to-date hydrological modelling, resulting in the overexploitation of water resources. Our findings show that water management policies have had a crucial role in the water volume changes of the Shortandy, emphasising the importance of future water management strategies for effective management of small endorheic lakes.

## Supporting information

S1 Fig Shortandy Lake bathymetry features. (a) Bathymetry map, (b) 3D model of the catchment, (c) Lake volume and lake area relationship.
(PDF)

S2 Fig Land cover map for the Shortandy catchment. The left figure shows the land cover map for the period of 1986–2009 and the right figure shows a land cover map from 2010–2016.
(PDF)

S3 Fig Historical changes in input and output variables of Shortandy Lake. (A) Comparison of monthly-averaged open water evaporation values between 2012–2016, where *E*_*O*_(water balance) is the average monthly open water evaporation estimated by the simplified Penman equation developed by Valiantzas and *E*_*O*_ (SSEBop) is the actual evapotranspiration values for the lake produced by Operational Simplified Surface Energy Balance model, (B) Comparison of monthly averaged evapotranspiration values where *E*_*act*_ is estimated using the FAO-56 Penman-Monteith in 1986–2016, Global-PET data for 1970–2000, and PET_Modis_ is potential evapotranspiration derived from MOD16A3GF for the period 2000–2016, (C) Ice-free dates and temperature dynamics, where dates are in Julian days, and *T*_*mean*_ is the mean air temperature deviation of April and May, (D) Mean annual snow water equivalent (SWE) and total annual snow, (E) Total duration of seasonal snowmelt in the forest and grassland, and the mean air temperature during the snowmelt season, (F) Comparison of total annual snowmelt runoff.
(PDF)

S4 Fig Estimation of groundwater flux using groundwater model (i). *V*_*b*.*m*_ is water volume at the beginning of the cold-season estimated by measured water levels; *V*_*e*.*m*_ is water volume at the end of the cold-season estimated by measured water levels.
(PDF)

S5 Fig Estimation of groundwater flux using groundwater model (ii). *V*_*begin*_ is the water volume of the lake estimated by measured water levels at the beginning of the year; *V*_*end*_ water volume estimated by measured lake level at the end of the year; I-O is the difference between input and output variables based on Eq 1.
(PDF)
