# Supplementary material for: Unravelling long-term impact of water abstraction and climate change on endorheic lakes: A case study of Shortandy Lake in Central Asia
Source: PLoS One. 2024 Jul 18;19(7):e0305721. doi: 10.1371/journal.pone.0305721 (PMC11257406; doi:10.1371/journal.pone.0305721)

S1 Fig. Shortandy Lake bathymetry features. (a) Bathymetry map, (b) 3D model of the catchment, (c) Lake volume and lake area (d) Lake level and lake area relationship.

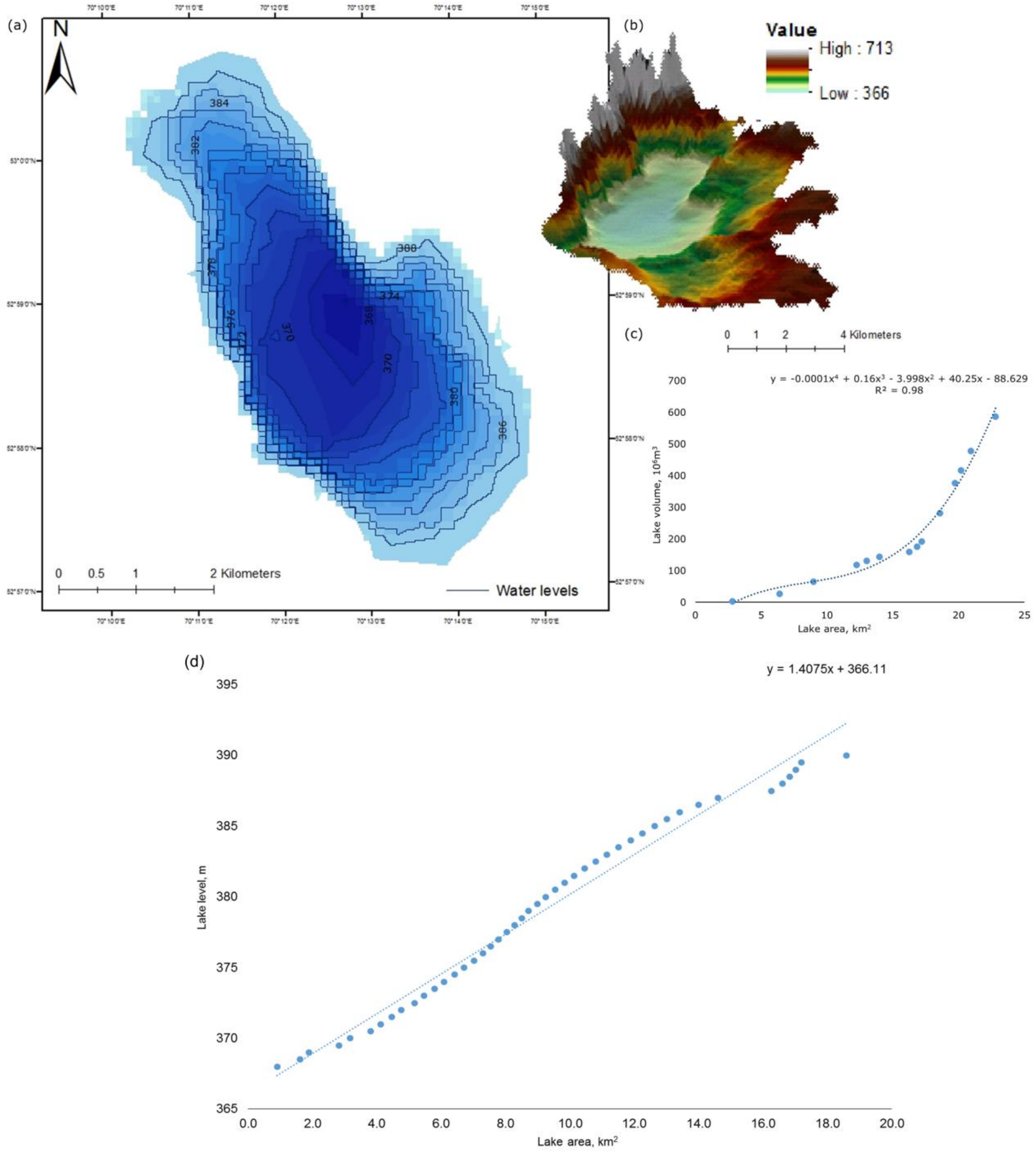

Supplement: S1 Fig — (a) Bathymetry map, (b) 3D model of the catchment, (c) Lake volume and lake area relationship. (PDF) [file pone.0305721.s001.pdf]
