# Supplementary material for: Unravelling long-term impact of water abstraction and climate change on endorheic lakes: A case study of Shortandy Lake in Central Asia
Source: PLoS One. 2024 Jul 18;19(7):e0305721. doi: 10.1371/journal.pone.0305721 (PMC11257406; doi:10.1371/journal.pone.0305721)

S2 Fig. Land cover map for the Shortandy catchment. The left figure shows the land cover map for the period of 1986-2009 and the right figure shows a land cover map from 2010-2016

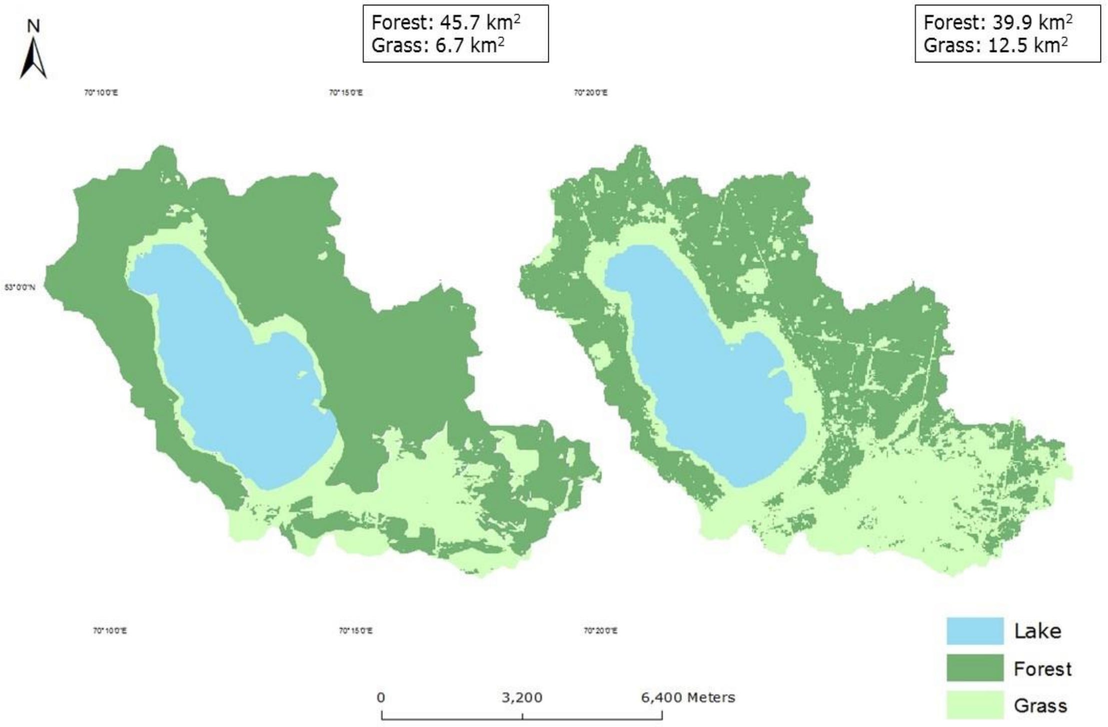

Supplement: S2 Fig — The left figure shows the land cover map for the period of 1986–2009 and the right figure shows a land cover map from 2010–2016. (PDF) [file pone.0305721.s002.pdf]
