# Supplementary material for: Unravelling long-term impact of water abstraction and climate change on endorheic lakes: A case study of Shortandy Lake in Central Asia
Source: PLoS One. 2024 Jul 18;19(7):e0305721. doi: 10.1371/journal.pone.0305721 (PMC11257406; doi:10.1371/journal.pone.0305721)

**S3 Fig. Historical changes in input and output variables of Shortandy Lake.** (A) Comparison of monthly-averaged open water evaporation values between 2012-2016, where $E_O$(water balance) is the average monthly open water evaporation estimated by the simplified Penman equation developed by Valiantzas and $E_O$(SSEBop) is the actual evapotranspiration values for the lake produced by Operational Simplified Surface Energy Balance model, (B) Comparison of monthly averaged evapotranspiration values where $E_{act}$ is estimated using the FAO-56 Penman-Monteith in 1986-2016, Global-PET data for 1970-2000, and $PET_{Modis}$ is potential evapotranspiration derived from MOD16A3GF for the period 2000-2016, (C) Ice-free dates and temperature dynamics, where dates are in Julian days, and $T_{mean}$ is the mean air temperature deviation of April and May, (D) Mean annual snow water equivalent (SWE) and total annual snow, (E) Total duration of seasonal snowmelt in the forest and grassland, and the mean air temperature during the snowmelt season, (F) Comparison of total annual snowmelt runoff.

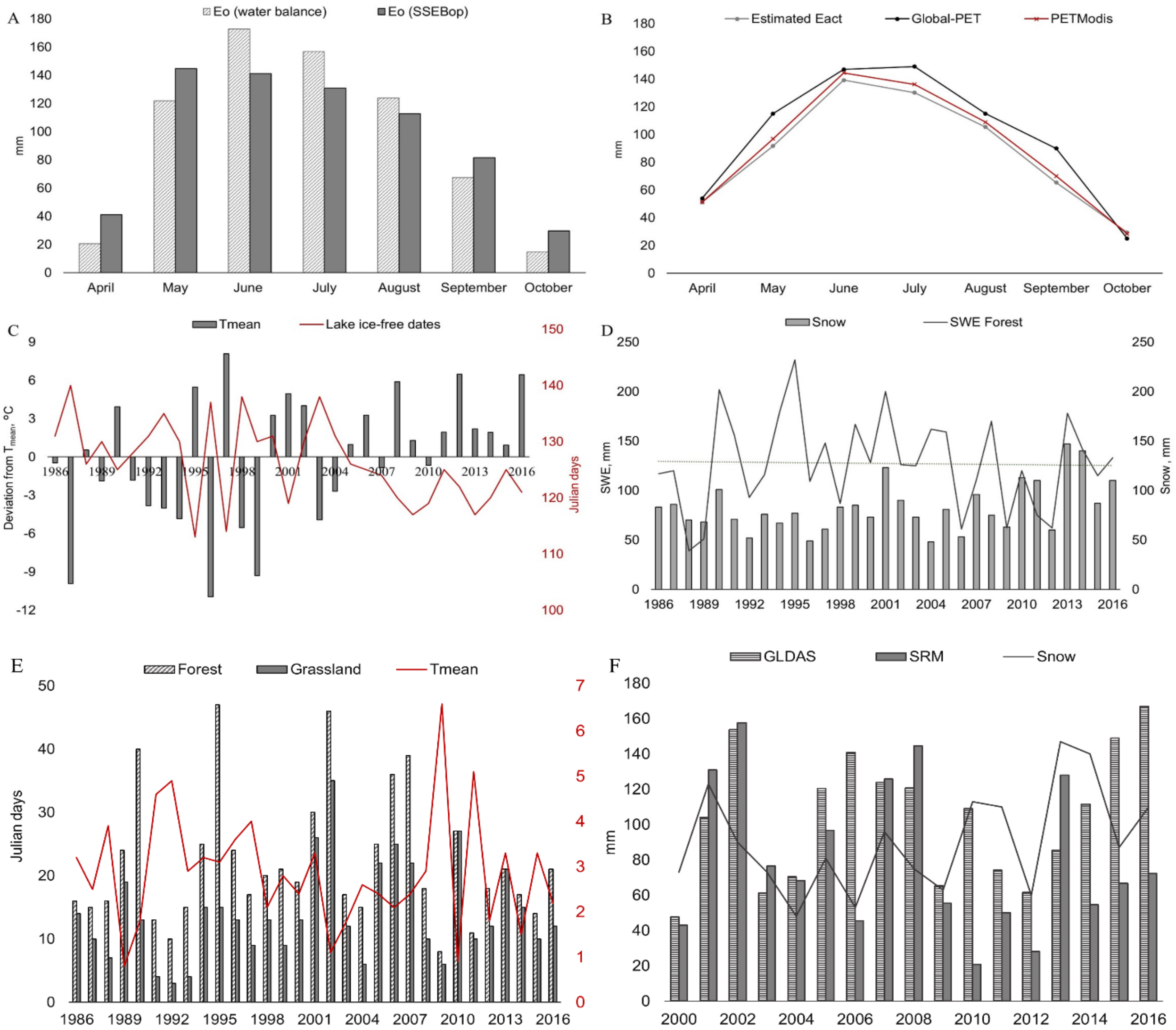

Supplement: S3 Fig — (A) Comparison of monthly-averaged open water evaporation values between 2012–2016, where EO(water balance) is the average monthly open water evaporation estimated by the simplified Penman equation developed by Valiantzas and EO (SSEBop) is the actual evapotranspiration values for the lake produced by Operational Simplified Surface Energy Balance model, (B) Comparison of monthly averaged evapotranspiration values where Eact is estimated using the FAO-56 Penman-Monteith in 1986–2016, Global-PET data for 1970–2000, and PETModis is potential evapotranspiration derived from MOD16A3GF for the period 2000–2016, (C) Ice-free dates and temperature dynamics, where dates are in Julian days, and Tmean is the mean air temperature deviation of April and May, (D) Mean annual snow water equivalent (SWE) and total annual snow, (E) Total duration of seasonal snowmelt in the forest and grassland, and the mean air temperature during the snowmelt season, (F) Comparison of total annual snowmelt runoff. (PDF) [file pone.0305721.s003.pdf]
