# Supplementary material for: Unravelling long-term impact of water abstraction and climate change on endorheic lakes: A case study of Shortandy Lake in Central Asia
Source: PLoS One. 2024 Jul 18;19(7):e0305721. doi: 10.1371/journal.pone.0305721 (PMC11257406; doi:10.1371/journal.pone.0305721)
Supplement: S4 Fig — Vb.m is water volume at the beginning of the cold-season estimated by measured water levels; Ve.m is water volume at the end of the cold-season estimated by measured water levels. (PDF) [file pone.0305721.s004.pdf]

S4 Fig. Estimation of groundwater flux using groundwater model (i). $V_{b.m}$ is water volume at the beginning of the cold-season estimated by measured water levels; $V_{e.m}$ is water volume at the end of the cold-season estimated by measured water levels.

Year	***Month***	$V_{b.m}$, **10^6 m^3**	$V_{e.m}$, **10^6 m^3**	$G_i - G_o$, **10^6 m^3**
**1986**				***-0.1***
	January	231.8	231.9	0.2
	February	231.9	232.1	0.2
	March	232.1	232.1	0
	November	228.6	228.4	-0.2
	December	228.4	228.1	-0.3
**1987**				***-0.3***
	January	228.1	228.1	0
	February	228.1	228.1	0
	March	228.1	228.1	0
	November	226.1	226.1	0
	December	226.1	225.8	-0.3
**1991**				***0.7***
	January	211.45	211.8	0.35
	February	211.8	211.8	0
	March	211.8	212.1	0.3
	November	204.6	204.6	0
	December	204.6	204.6	0
**1992**				***-0.5***
	January	204.6	204.3	-0.3
	February	204.3	204	-0.3
	March	204	203.9	-0.1
	November	200.0	200.0	0
	December	200.0	200.2	0.2
**1993**				***-0.6***
	January	200.2	200.2	0
	February	200.2	200.0	-0.2
	March	200.0	200.0	0
	November	202.6	202.3	-0.3

	December	202.3	202.3	0
**1994**				***0.3***
	January	202.4	202.1	-0.3
	February	202.1	202.1	0
	March	202.1	202.1	0
	November	202.5	202.5	0
	December	202.5	203.2	0.7
**1995**				***0***
	January	203.2	203.2	0
	February	203.2	203.2	0
	March	203.2	203.2	0
	November	197.3	197.3	0
	December	197.3	197.3	0
**1996**				***0.2***
	January	197.3	197.5	0.2
	February	197.5	197.5	0
	March	197.5	197.5	0
	November	195.1	195.1	0
	December	195.1	195.1	0
**1997**				***-1.2***
	January	197.5	197.5	0
	February	197.5	197.5	0
	March	197.5	197.5	0
	November	188.4	187.2	-1.2
	December	187.2	187.2	0
**1998**				***0***
	January	187.2	187.2	0
	February	187.2	187.2	0
	March	187.2	187.2	0
**2003**				***-0.2***
	January	189.2	189.2	0
	February	189.2	189.0	-0.2
	March	189.0	189.0	0

	November	185.5	185.0	-0.5
	December	185.0	185.4	0.5
**2004**				***-1.0***
	January	185.4	185.2	-0.2
	February	185.2	185.2	0
	March	185.2	185.2	0
	November	179.8	179.0	-0.8
	December	179.0	179.0	0
**2005**				***1.0***
	January	179.0	179.3	0.3
	February	179.3	179.5	0.2
	March	179.5	179.5	0
	November	181.7	181.7	0
	December	181.7	182.2	0.5
**2006**				***1.1***
	January	182.2	182.9	0.7
	February	182.9	183.1	0.2
	March	183.1	183.3	0.2
	November	182	182	0
	December	182	182	0
**2007**				***-1.6***
	January	182	182.2	0.2
	February	182.2	182.7	0.5
	October	183.6	182.8	-0.8
	November	182.8	182	-0.8
	December	182	181.3	-0.7
**2008**				***-0.8***
	January	181.3	181.3	0
	February	181.3	181.6	0
	March	181.6	181.6	0
	November	178.8	178.3	-0.5
	December	178.3	178	-0.3
**2009**				***0.3***

	January	178	178	0
	February	178	178.3	0.3
	March	178.3	178.3	0
	November	176.8	176.6	-0.2
	December	176.6	176.8	0.2
**2010**				***2.1***
	January	176.8	176.3	0.5
	February	176.3	177.2	0.9
	March	177.2	177.7	0.5
	December	172.1	172.3	0.2
**2011**				***1.0***
	January	172.3	172.6	0.3
	February	172.6	172.8	0.2
	March	172.8	173.1	0.3
	November	171.6	171.6	0
	December	171.6	171.8	0.2
**2012**				***1.0***
	January	171.8	171.8	0
	February	171.8	172	0.2
	March	172	172	0
	November	167.6	167.6	0
	December	167.6	168.4	0.8
**2013**				***1.6***
	January	168.4	168.7	0.3
	February	168.7	169	0.3
	March	169	169.7	0.7
	December	171.4	171.7	0.3
**2014**				***0.8***
	January	171.7	172.2	0.5
	February	172.2	172.7	0.5
	March	172.7	173	0.3
	October	173.1	172.1	-1.0
	November	172.1	172.3	0.2

	December	172.3	172.6	0.3
**2015**				***1.3***
	January	172.6	173.1	0.5
	February	173.1	173.4	0.3
	March	173.4	173.7	0.3
	November	172.4	172.1	-0.3
	December	172.1	172.6	0.5
**2016**				***2.0***
	January	172.6	173.1	0.5
	February	173.1	173.4	0.3
	March	173.4	173.6	0.2
	November	173.9	174.2	0.3
	December	174.2	172.9	0.7
**Average**				***+0.29***
