# Supplementary material for: Unravelling long-term impact of water abstraction and climate change on endorheic lakes: A case study of Shortandy Lake in Central Asia
Source: PLoS One. 2024 Jul 18;19(7):e0305721. doi: 10.1371/journal.pone.0305721 (PMC11257406; doi:10.1371/journal.pone.0305721)
Supplement: S5 Fig — Vbegin is the water volume of the lake estimated by measured water levels at the beginning of the year; Vend water volume estimated by measured lake level at the end of the year; I-O is the difference between input and output variables based on Eq 1. (PDF) [file pone.0305721.s005.pdf]

S5 Fig. Estimation of groundwater flux using groundwater model (ii). V_{begin} is the water volume of the lake estimated by measured water levels at the beginning of the year; V_{end} water volume estimated by measured lake level at the end of the year; I-O is the difference between input and output variables based on Eq.1.

**Year**	V_{begin}, **10^6m^3**	V_{end}, **10^6m^3**	$V_{begin} - V_{end}$, **10^6m^3**	I-O, **10^6m^3**	$G_i - G_o$, **10^6m^3**
**1986**	231.6	228.3	-4.5	-3.4	-0.1
**1987**	228.3	226.0	-2.3	-2.3	0.1
**1991**	211.4	204.6	-6.8	-6.8	1.0
**1992**	204.6	200.2	-4.5	-4.5	0.2
**1993**	200.2	202.0	1.8	1.8	-0.6
**1994**	202.0	202.8	0.8	0.8	-1.1
**1995**	202.8	197.4	-5.4	-5.1	-0.6
**1996**	197.4	195.4	-2	1.5	-0.3
**1997**	195.4	188.7	-6.7	-6.7	-1.0
**2003**	189.2	185.5	-3.7	-3.7	0.3
**2004**	185.5	180.1	-5.4	-5.4	-1.0
**2005**	180.1	182.2	2.0	2.0	1.5
**2006**	182.2	182.0	-0.2	-0.2	1.5
**2007**	182.0	182.7	0.7	0.7	-1.5
**2008**	182.7	178.0	-4.7	-4.7	-1.4
**2009**	178.0	177.1	-0.9	-0.9	-1.2
**2010**	177.1	172.1	-5.1	-5.1	2.3
**2011**	172.1	171.6	-0.5	-0.5	1.4
**2012**	171.6	168.3	3.3	-3.3	1.5
**2013**	168.3	171.6	3.3	3.3	0.1
**2014**	171.6	173.1	1.5	1.5	1.2
**2015**	173.1	172.6	-0.5	-0.5	1.4
**2016**	172.6	174.9	1.9	1.9	3.3
**Average**					**+0.*16***
